# Digital media balance and mental health: effects of a school-based program

**DOI:** 10.1186/s13034-026-01131-3

**Published:** 2026-07-10

**Authors:** Mona Guath, Sissela B. Nutley, Lisa B. Thorell

**Affiliations:** 1 Department of Psychology, Uppsala University, Box 1225, 751 42 Uppsala, Sweden; 2 Department of Clinical Neuroscience, Karolinska Institutet, Nobels väg 9, 171 77 Stockholm, Sweden

**Keywords:** Digital balance, School-based program, Mental health, Adolescence, SEL, Psychological well-being

## Abstract

**Background:**

Adolescent mental health problems are rising globally, with problematic digital media use identified as an important risk factor. Universal school-based interventions that integrate Social and Emotional Learning (SEL) with digital balance strategies may promote resilience and reduce risk. This study evaluated On the Inside, a classroom-based SEL and digital balance program.

**Methods:**

A total of 750 students aged 13–15 years from eight schools (two control schools) participated. Surveys were completed at baseline (T1), post-intervention (T2), and, for a subsample, one year later (T3). The program comprised five video-based sessions delivered by teachers. Outcomes included psychological distress (PHQ-4), problematic social media use (GSMQ-9), and adaptive behaviors. Data were analyzed using multilevel mixed models to account for clustering by school.

**Results:**

Relative to controls (n = 193), students in the intervention group (n = 557) reported reductions in psychological distress, problematic social media use, multitasking, and avoidance, alongside increased use of adaptive strategies such as distancing phones during study and challenging negative thoughts. Several effects, including reduced distress and avoidance, and sustained strategy use, remained at one-year follow-up. No significant effects were observed for sleep, help-seeking, or breathing strategies.

**Conclusions:**

This universal, teacher-delivered program integrating SEL and digital balance was feasible and produced small but clinically meaningful improvements in adolescent distress and related behaviors, with some effects sustained after one year. These findings support the potential of school-based interventions to address digital media-related risks and promote mental health at a population level. Further research should refine program components, particularly for sleep and screen time, and examine cost-effectiveness to inform broader implementation.

**Supplementary Information:**

The online version contains supplementary material available at 10.1186/s13034-026-01131-3.

## Introduction

Adolescence has long been recognized as a particularly challenging developmental stage, during which mental health issues can emerge. Over the past decade, the number of children and adolescents seeking health care for mental health concerns in high-income countries has increased dramatically [1, 2]. Several factors contribute to the risk of developing poor mental health, including genetic predisposition, life circumstances, heightened awareness of global concerns, and daily habits [3]. For example, only 20% of adolescents engage in sufficient daily physical activity [4], and in many countries, fewer than 50% meet recommended sleep guidelines [5–8]. Increased use of digital media has also been implicated, as the rise in adolescent mental health problems coincides with the widespread adoption of smartphones and social media apps [1, 2, 9].

Although a variety of school-based interventions have been implemented to promote mental health or digital addictions, there is still a lack of interventions focusing on both digital media balance and social emotional learning (SEL). In addition, there is a lack of follow-up studies investigating whether effects persist over time. Therefore, the present study aimed to evaluate a novel school-based intervention that addresses both digital media balance and mental health promotion, assessing outcomes immediately after the intervention and at a one-year follow-up.

### Digital media use and mental health issues

Digital media has become fully integrated into adolescents’ daily lives, with both positive and negative consequences. In 2023, nearly half of adolescents reported being online “almost constantly,” compared with 24% in 2014 [10]. Objective tracking studies indicate that 41% of adolescents use their smartphones more than five hours daily, with 9% exceeding ten hours per day [11]. Excessive social media use has been associated with heightened mental health risks, particularly for girls, through mechanisms such as social comparison, exposure to unrealistic beauty standards [12], negative online interactions such as bullying, sexting, grooming [13, 14], and displacement of protective behaviors such as sleep [15–19]. Conversely, digital interactions can have beneficial effects; for example, texting with friends has been shown to improve mood after stressful events [20].

Importantly, while excessive screen time can have serious displacement effects, research consistently shows that problematic or addictive patterns of social media use show stronger associations with mental health problems than overall time spent online [21, 22]. Data from the Health Behavior in School-aged Children (HBSC) study across 44 countries indicate that the prevalence of addictive-like or problematic social media use increased from 7% in 2018 to 11% in 2022 [23]. Problematic use can be further distinguished into “heavy involvement” and “negative consequences.” The negative consequences component, which includes displacing other interests, escaping reality, or lying about usage, is more strongly associated with mental health problems than the heavy involvement component, which includes being preoccupied or showing signs of withdrawal or tolerance [24].

The context and motivations for use are also of importance; using social media for escape, achieving higher social status, or interacting with strangers is associated with problematic use, whereas interacting with established friends, or using social media for entertainment or relaxation, is less troublesome [25–27]. Using social media to escape negative thoughts and feelings appears especially problematic, even after controlling for the overlap between different types of motives and screen time [28]. This emphasizes the need to teach adolescents additional strategies for regulating negative feelings. Finally, some children, such as those with Attention-Deficit/Hyperactivity Disorder (ADHD; [29, 30]), Autism Spectrum Disorder (ASD; [31]), and youth with pre-existing mental health problems [32], are particularly vulnerable to developing problematic digital media use or experiencing more severe consequences from excessive or addictive use [27]. Reducing digital media use may therefore be of even greater importance for these children [33]. These complexities are captured in the recently proposed “Digital Media-use Effects in children and adolescent model” [34]. This model includes four different parts: a) psychological mechanisms relevant to specific situations of digital media use (e.g., affective and cognitive mechanisms and media use behavior), b) proximal factors that define the specific media-use situation (e.g., characteristics of the media, the situation, or availability of alternatives), c) distal factors such as individual traits, as well as the social, regulatory and cultural context, and d) beneficial or harmful outcomes, distinguishing between short-term effects (e.g., mood changes) and long-term outcomes (e.g., mental health development). Taken together, the rising prevalence of problematic use and evolving understanding of factors that support healthy digital media habits underscore the need for prevention efforts and interventions that promote emotion regulation and digital media balance.

### Digital media use and distractions from healthy habits

School-aged children face daily cognitive challenges, including sustaining attention, filtering distractions, and learning new information. Many adolescents frequently multitask with digital media while studying, for example, chatting or watching videos, which incurs attentional switching costs, prolongs task completion, and can impair academic performance [35, 36]. While some of this multitasking occurs on school computers, smartphone use for social media, video streaming, or listening to music (especially with lyrics) is also common and can negatively affect learning [37]. Objective tracking indicates that half of adolescents receive over 200 notifications daily, further disrupting attention [11].

Longitudinal studies suggest that habitual multitasking while studying can impair attentional control [36, 38], with potential detrimental effects on cognitive development [39]. Digital media habits such as keeping a smartphone within reach during study sessions have been shown to reduce attention and working memory capacity, thereby impairing learning [40]. Similarly, the engaging features of smartphones and social media can lead to prolonged scrolling, displacing sleep, exercise, study time, and family or social interactions. Understanding how digital apps capture attentional resources, often driven by business models that monetize user time, may be essential to motivate behavioral change.

### When and how to intervene

The prevalence of mental disorders nearly doubles between ages 5–9 and 10–14 (rising to 12%) and then remains relatively stable into adulthood [41], suggesting that early adolescence may be a critical period for preventive interventions. This developmental stage coincides with substantial increases in digital media use and a gradual withdrawal of parental engagement, highlighting the importance of establishing and maintaining healthy habits during this stage. Given that mental well-being and digital media balance are important for nearly all children, and some health behaviors require normative shifts, school-based programs are an effective format for large-scale delivery.

Regarding how to intervene, a variety of school-based interventions have been evaluated with mixed results. For example, positive psychology programs have improved well-being in some studies [42], whereas well-being interventions have generally not yielded significant effects [43]. Mental health literacy programs have produced small improvements in help-seeking intentions [44], and daily mindfulness exercises have shown benefits for certain subgroups, such as girls in primary school or secondary school students with higher emotional difficulties [45]. Careful consideration of which abilities, behaviors, and strategies are most relevant to mental health, and the selection of appropriate outcomes, is therefore essential when designing and evaluating interventions.

SEL programs have generally been successful in reducing emotional distress and improving SEL skills compared to control groups [46]. SEL programs can be based on various frameworks, with the Collaborative for Academic, Social, and Emotional Learning’s (CASEL) model including the following five competency domains: self-awareness, self-management, responsible decision-making, social awareness, and relationship skills, being particularly influential [47, 48]. Successful programs often follow the SAFE criteria: Sequenced (connected and coordinated activities), Active (active learning), Focused (developing personal and then social skills), and Explicit (targeting specific SEL skills) [49].

Recent meta-analyses confirm that adherence to SAFE criteria is important and that programs are often more effective when delivered by teachers who already have established relationships with students, rather than external personnel [46]. However, this approach requires adequate training, time, and resources to support teachers’ development of SEL competencies. Parents have also expressed a desire for schools to emphasize digital balance and provide psychoeducation to both students and caregivers [50], potentially further increasing demands on teachers. Conversely, some evidence suggests that programs delivered by clinicians, programs targeting anxiety (compared with depression), and those incorporating cognitive-behavioral techniques produce stronger effects [51].

A limited number of school-based programs have specifically addressed digital media issues, with most focusing on preventing gaming or internet addiction (for a recent review, see [52]). These programs typically aim to enhance self-control, promote healthy internet habits, reduce overall internet use, and prevent addictive behaviors. While most studies reported reductions in addictive use, the target skills and strategies varied. Several challenges have been identified, including a lack of consensus on the definition of internet addiction and appropriate measurement approaches, which complicate the interpretation of results [53]. Programs that actively involve parents, targeted at-risk youth, incorporate therapy-based approaches, or are delivered externally have generally been the most successful in reducing problematic digital technology use [52]. Interventions aimed at reducing screen time have also shown greater effectiveness when delivered by researchers [54], likely due to the rapidly evolving digital knowledge required to teach these skills. However, reliance on external delivery creates practical challenges for schools seeking to implement programs consistently. These considerations underscore the potential value of a scientifically grounded, video-based program that delivers up-to-date expertise on digital balance and cognitive-behavioral strategies, while allowing teachers to facilitate and adapt content in the classroom. Such an approach would leverage teachers’ established relationships with students, minimize additional workload, and ensure accurate knowledge transfer at scale.

### Combined intervention for digital media balance and SEL

The negative effects of digital media extend beyond addictive use and include bullying, social comparison, exposure to unrealistic beauty standards, grooming, and displacement of health-protective behaviors. These wide-ranging impacts underscore the need for a broader approach than merely reducing time spent online. Problematic digital media use and mental health issues are interrelated, often forming reinforcing spirals, and should therefore not be addressed in isolation. Key factors to target in preventing addictive-like use include managing escapism, self-identity, attentional control, emotion regulation, and coping with stress [55, 56]. Preventive efforts may also benefit from incorporating broader health-promoting strategies [18, 57] or whole-school initiatives, which have demonstrated reductions in cyberbullying [58].

In addition, resistance to digital habit formation may further require basic knowledge of interaction design features in digital apps to identify and manage their influence. Evidence suggests that behavioral strategies can effectively support digital media balance. For example, a randomized controlled trial that provided participants with ten strategies to reduce screen time, such as disabling notifications, hiding social media apps, or turning off Face ID, yielded positive effects on both psychological well-being and screen time [59]. Simple environmental modifications, such as placing the phone further away during study periods, may also foster better learning habits and academic performance [27].

A final key aspect is that discussions about screen time and the potential risks of digital media are often conveyed by adults as restrictions, rules, or bans. This approach can elicit a naturally defensive stance among adolescents receiving messages about digital well-being. Previous research has shown benefits of using media and interactive exercises in intervention programs for adolescents [60]. While more research is needed, employing drama and third-party characters to illustrate common challenges has been shown to reduce anxiety about self-disclosure in the classroom, decrease stigma, and promote engagement [61]. This may be particularly effective during adolescence, when students naturally seek more independence from adult control. However, it remains unknown whether this approach can be successfully implemented in an intervention for adolescents targeting improvements in mental health outcomes, behaviors, and digital media management.

### Current study

The research presented above indicates a need for a comprehensive intervention that combines basic mental health promotion, including SEL content, with strategies for digital media balance and practical tools to achieve behavioral change. The aim of this study was to evaluate whether a combined digital media balance and SEL intervention delivered through classroom films and activities positively impacts adolescents’ mental health outcomes, behaviors, and use of strategies compared to a control group.

### Hypotheses

H1: The intervention group will demonstrate more favorable outcomes than the control group on mental health-related measures, including psychological distress (Patient Health Questionnaire; PHQ-4 [62], primary outcome), health-related quality of life (KIDSCREEN-10 [63]), and problematic social media use (Gaming and Social Media Questionnaire, GSMQ-9 [21]).

H2: The intervention group will exhibit more favorable health behaviors compared to the control group, as measured by sleep duration (hours/night), avoidant behaviors, multitasking habits, problematic online interactions, and time spent on smartphones.

H3: The intervention group will report increased use of strategies compared to the control group, including greater help-seeking behaviors (assessed via the General Help Seeking Questionnaire [GHSQ, 64]), more frequent use of breathing techniques, relocating smartphones while studying, challenging thought distortions, and adjusting smartphone notification settings.

## Method

### Participants

Adolescents were recruited from all six public schools in a medium-sized Swedish city for the intervention group, and from two schools in demographically similar areas for the control group during the autumn term of 2022. All students participating in lessons on the day of the survey were eligible, and informed consent was obtained from 946 of 1,057 enrolled adolescents (111 were absent or did not consent). At the second measurement time point (T2), 557 students in the intervention group (82% of the 682 baseline participants) and 193 students in the control group (73% of 264 baseline participants) were successfully matched to their baseline measurements (T1). The higher attrition in the control condition was due to one of the schools rotating class-belonging during the school year, making it impossible to match individual data between T1 and T2 for some children. Participants were aged 13–15 years (grades 7 and 8). Demographic characteristics, including housing type (villa, owned apartment, or rental) as a proxy for socioeconomic status, and self-reported diagnostic status, were collected (see Table 1). A follow-up at one-year post-intervention (T3) was conducted for a subset of the intervention group (*n* = 188, of whom 122 provided complete data across all three time points). The control schools began implementing the school program after T2 and could therefore not be included at T3. Teachers (*n* = 40) in the intervention schools completed surveys at the end of the school year.

**Table 1:** Study participant demographics

Demographic information		Intervention *n* (%)	Control *n* (%)	*p-*value
Sample size	T1, baseline	682 (100)	264 (100)	
	T2, end of intervention	557 (81.7)	193 (73.1)	
	T3, 12 months after T2	122 (64.9)	a	
Gender	Girls	271 (48.6)	95 (49.2)	0.10
	Boys	274 (49.2)	94 (48.7)	
	Other/prefer not to answer	12 (2.2)	2 (1.0)	
Housing	Villa	376 (67.5)	123 (63.7)	< 0.001
	Owned apartment	52 (9.3)	46 (23.8)	
	Rental apartment	129 (23.2)	24 (12.4)	
Age	13–14 years, grade 7	418 (75.1)	103 (53.4)	< 0.001
	14–15 years, grade 8	139 (24.9)	90 (46.6)	
Diagnosis	ADHD/ASD/depression/anxiety/other	129 (23.1)	38 (19.7)	0.23

^a^ The control group could not be followed until T3, as they had started participating in the intervention at that time

### Materials

#### Intervention

The intervention consisted of five interactive video modules, each following a consistent structure: dramatized scenes depicting a common mental health challenge, automated prompts for classroom discussion, scientific explanations from experts, and exercises applying self-help strategies initially to the characters in the videos. The content framework was developed collaboratively by researchers in cognitive neuroscience and psychology, drama writers, and reviewed by licensed psychologists. The videos and materials were produced by the nonprofit organization Arts & Hearts and collectively titled “On the Inside” (“Det syns inte” in Swedish).

As described in the introduction, it is important to encourage student engagement and motivation for change. The program evaluated in this study was developed with these principles in mind. High-quality films featuring third-party characters were used to depict common problems related to digital habits and mental health struggles, after which experts provided scientific explanations to the scene. Teacher-led discussions and exercises to practice skills complemented these video materials, combining social learning with standardized knowledge transfer to ensure consistent quality. Recognizing that today’s youth are accustomed to high-quality entertainment and information, the program integrated scientific explanations, expert interviews, and relatable role models demonstrating actionable strategies. By initially helping characters solve problems before applying solutions to themselves, the program aimed to reduce defensive responses and facilitate adoption of mental health strategies and behavioral changes.

During the school year prior to the study, the program was piloted with 65 other teachers and 618 adolescents at participating schools, after which minor adjustments were made to exercises, discussion questions, and instructions. There was no overlap between the students and teachers participating in the pilot and the main study. The five modules addressed common challenges related to digital habits and mental health: (1) healthy habits, (2) performance anxiety, (3) focus time, (4) shaping thoughts and feelings, and (5) habit-forming digital design. Each module included content for up to three sessions across the academic year, with teachers encouraged to deliver at least one session per module.

**Fig. 1:**
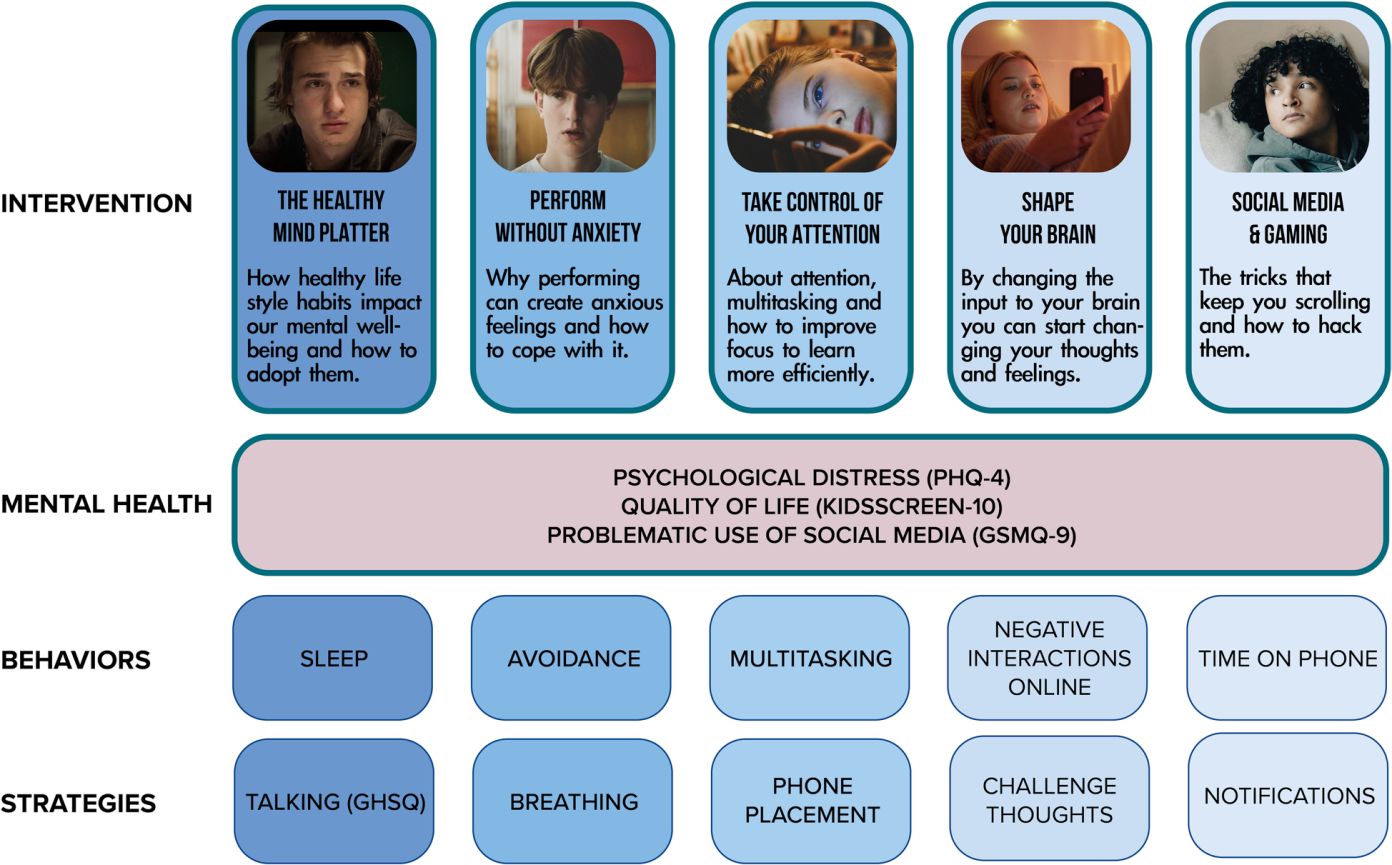
Framework for the intervention “On the inside”. Each module’s messaging corresponds to the hypothesized levels of change for (1) mental health related outcomes, (2) behaviors, and (3) strategies

In addition, short parent-facing films summarized the key messages from each module and provided guidance on supporting their adolescents at home. Teachers were encouraged to share these with parents after each classroom module. The key content and theoretical framework of each module are summarized in Fig. 1, with a detailed description provided in Table 1 of the Online Appendix.

#### Measures

At baseline (T1) and immediately post-intervention (T2), adolescents completed a 74-item questionnaire covering demographic information, mental health, digital media use, behaviors, and strategies. The intervention group completed the same questionnaire again at a one-year follow-up (T3). Teachers reported the number of completed intervention modules, which was used in the analysis. A summary of the measures is provided in Table 2 of the Online Appendix.

### Mental health related outcomes

Psychological distress was assessed using the short Patient Health Questionnaire (PHQ-4; 62), which includes two items on anxiety and two items on depressive symptoms experienced over the past two weeks. Participants responded on a four-point scale ranging from 0 (not at all) to 3 (daily), and a sum score was calculated for analysis. Health-related quality of life was measured using the KIDSCREEN-10 [63], which assesses well-being over the past week, including energy levels and relationships with friends and parents. Items were rated on a five-point scale from 1 (never) to 5 (always), and a sum score was used in the analyses. Symptoms of social media addiction were assessed with the Gaming and Social Media Questionnaire (GSMQ-9; 21), focusing on the subscale of negative consequences, which has been shown to be most predictive of mental health issues [24]. Participants indicated their agreement with statements such as “I use social media as a way to escape reality” and “My use of social media has had negative consequences for me” on a scale from 0 (strongly disagree) to 4 (strongly agree), and the mean score was used in analyses. This will be referred to as problematic use of social media.

### Behaviors

Behaviors and habits related to mental health were assessed using single-item questions developed and pilot-tested for this study. Sleep was measured by asking participants how many hours they typically sleep on a weekday, with response options ranging up to 10 h. Smartphone use was assessed by asking how many hours participants typically spend on their phones on a weekday, excluding school-related use, with responses ranging from 0 to more than 7 h per day. Multitasking was assessed with the item “How often do you study with media on, that isn’t related to the task (e.g., chatting, streaming videos, or listening to music with lyrics)?” Responses ranged from 0 (never/almost never) to 4 (always/almost always). Avoidant behaviors were measured by the statement “I usually avoid doing the things I worry about (e.g., raise my hand, talk in front of others),” with responses from 1 (strongly disagree) to 5 (strongly agree). Negative online interactions were assessed by summing the number of occurrences in the past month of behaviors such as sending or receiving mean comments online, feeling pressured to send nudes, or sending nudes to someone outside a relationship.

### Strategies

Mental health-related strategies were assessed using single-item questions developed for the study, except for talking about personal or emotional problems, which was measured using the General Help-Seeking Questionnaire (GHSQ; 64). Participants reported the number of people they had spoken with about personal or emotional problems over the past three weeks, which was summed to produce a total score. Phone placement during study time was assessed with response options including “right next to me,” “out of reach,” or “in a different room.” Other strategies included challenging thought distortions, managing smartphone notifications, and practicing breathing techniques. For example, participants responded to the statements “I try to challenge my negative thoughts and think in a different way,” “I have specific settings for which apps I receive notifications from,” and “I usually breathe slow and deep breaths when I want to calm myself” on a five-point Likert scale ranging from 1 (strongly disagree) to 5 (strongly agree).

### Teacher ratings

Teachers rated the perceived benefits of the program using five questions that targeted the main focus areas. For example, teachers were asked to rate if the program had been a valuable resource for addressing… “how mobile phones can affect sleep”, or “how to achieve a better digital media balance”. The questions were rated on a 5-point scale using the following response options: “strongly disagree” (1), “disagree” (2), “neither agree or disagree” (3), “agree” (4), and “strongly agree” (5). Teachers also rated satisfaction with the program using the same scale (e.g., “I would recommend the program to a colleague” and “I think that the program is relevant for the students”). Teachers were also asked to report any adverse events during program administration and any potential negative experiences among their students. Due to an undetected error in the survey (n = 14) and failure to finish the survey (n = 7), complete teacher ratings of satisfaction were unfortunately only available for 19 (47.5%) of the sample while incidents of adverse effects were collected from all (*n* = 40).

### Socioeconomic status

Socioeconomic status (SES) was indicated using housing type (villa, owned apartment, or rental) [65].

### Procedure

Six schools implemented the classroom-based program “On the Inside” over one school year, while two schools continued with their usual curriculum and served as the control group. The schools had independently decided to adopt the intervention prior to the study invitation, and students perceived the program as part of their regular education. The intervention sessions were most commonly led by the main teacher (72%) or by a member of the school healthcare team (28%). Information about participating in the study was sent to parents and students through the schools’ digital platforms. The survey was distributed digitally during a lesson, and completing it required approximately 10–15 min. Students who agreed to participate used a unique code to link their responses across time points, ensuring anonymity, as the researchers had no information that could identify individual participants. In addition, teachers in the intervention group were encouraged to share a link to the parent-facing films, one for each program module, and 63% reported doing so.

### Ethical considerations

The study procedures were carried out in accordance with the Declaration of Helsinki, and the Swedish Ethical Review Authority approved the study (#2022–03610-01–322702). All participants and their caregivers were informed about the study, and all respondents provided informed consent.

### Design and analyses

We used multilevel models to evaluate the three outcome domains targeted by the intervention: mental health-related outcomes, behaviors, and strategies. The study employed a nested mixed design, with group (intervention vs. control) as the between-subjects variable and measurement point (T1 vs. T2) as the within-subjects variable. Participants were nested within classrooms and schools. Due to differences in classroom organization in one of the control schools, the final models included two levels: adolescents nested within schools. A subset of the intervention group was assessed a third time (T3), approximately 12 months after the second assessment; this measurement point could therefore be analyzed only within the intervention group among adolescents who completed all three assessments. Follow-up at T3 was limited to grade 7 as data collection was deemed to interfere with final exams or a too heavy student workload in the higher grade. The analysis plan was preregistered prior to conducting the analyses (https://osf.io/j5dne/).

For each outcome domain, a null model and a full multi-level model, including all predictors and covariates listed in Table 2 of the Online Appendix, were estimated. As there were significant group differences between the intervention and control groups (see Table 1) for both SES (i.e., 3-level housing variable) and age (i.e., grade), these variables were also included as covariates. We also included gender as a covariate because previous studies have found gender differences both in digital media use and its association with mental health problems [66]. In total, three models were fitted for each type of analysis, and effect sizes for significant treatment effects were reported as partial R^2^, with cutoffs for small (0.01), medium (0.09), and large (0.25) effects, which are notably lower than Cohen’s d ([67]; see [68] for further explanation). To assess the robustness of our conclusions, we applied a Benjamini–Hochberg false discovery rate (FDR) adjustment across all time x group tests (13 outcomes × 1 interaction each), controlling FDR at 5% [69]. The results are included in all tables with an interaction effect in the Appendix.

Most outcome variables were measured on Likert scales, which were often not normally distributed. For these ordinal responses, cumulative link mixed models (CLMMs) were employed with a cumulative logit link to account for the ordered nature of the data. When Likert-scale variables were approximately normally distributed, linear multilevel models were used. Count variables were modeled with generalized linear mixed models (GLMMs) with Poisson distribution. Variables with excess zeroes, such as negative online interactions and help-seeking (GHSQ), were analyzed using zero-inflated models, which treat zero observations separately from nonzero values, thereby better accommodating their distribution (see [70] for further discussion). Finally, the GSMQ negative consequences subscale, a continuous variable with a right-skewed distribution, was modeled using GLMMs with a Gamma distribution. The coefficients in all models with a logit link are reported as incidence ratios (i.e., exponentiated) for ease of interpretation.

A significant group × time interaction in the models was used to evaluate whether the respective hypotheses were supported. For interaction effects favoring the intervention group, follow-up analyses assessed whether these effects were sustained one year after the intervention. Models with normally distributed, count, or Gamma-distributed outcomes were estimated using restricted maximum likelihood with the lme4 package [71] in R version 4.4.1 [72]. Ordinal regression models were analyzed using the ordinal package [73], while zero-inflated models were fitted using the glmmTMB package [74]. Additional analyses examining the relationship between the number of modules completed in the classroom and mental health outcomes were treated as sensitivity analyses. These analyses provided further insight into how the degree of intervention implementation was related to the outcomes. Only significant treatment effects are presented in the Results section. For the complete model outputs, including intraclass correlation coefficients (ICCs) and conditional R^2^ values for the full models, we refer readers to the Appendix.

## Results

The descriptive statistics for the continuous outcome variables by group and measurement point are summarized in Table 2.

**Table 2:** Mean and standard deviations for the intervention group and the control group for the three time points

Measure	Group	T1, M (SD)	T2, M (SD)	T3, M (SD)
Psychological distress	Control	3.6 (2.6)	3.8 (2.7)	
	Intervention	3.5 (2.5)	2.7 (2.3)	2.7 (2.4)
Quality of life	Control	35.1 (4.2)	35.0 (4.2)	
	Intervention	35.8 (3.8)	35.5 (4.2)	35.3 (2.8)
Problematic social media use	Control	1.6 (0.7)	1.6 (0.7)	
	Intervention	1.7 (0.8)	1.5 (0.7)	1.6 (0.6)
Sleep average (h)	Control	7.9 (1.0)	7.8 (1.0)	
	Intervention	7.9 (1.0)	7.9 (1.0)	7.9 (1.0)
Multitasking	Control	2.9 (1.1)	3.0 (1.2)	
	Intervention	2.9 (1.3)	2.4 (1.2)	2.5 (1.3)
Avoidance	Control	2.9 (1.2)	2.9 (1.2)	
	Intervention	2.8 (1.3)	2.5 (1.3)	2.4 (1.2)
Negative online interactions	Control	1.1 (3.7)	1.3 (4.9)	
	Intervention	1.0 (3.2)	0.6 (2.2)	1.0 (3.0)
Smartphone daily (h)	Control	3.8 (1.6)	4.1 (1.6)	
	Intervention	3.9 (1.6)	3.9 (1.6)	4.1 (1.7)
Notification settings	Control	3.1 (1.4)	3.2 (1.4)	
	Intervention	3.0 (1.4)	3.1 (1.4)	3.3 (1.4)
Breathing strategies	Control	2.7 (1.2)	2.7 (1.2)	
	Intervention	2.6 (1.3)	2.9 (1.3)	2.8 (1.3)
Talk about problems	Control	1.1 (1.3)	1.1 (1.2)	
	Intervention	1.2 (1.5)	1.2 (1.5)	1.2 (1.4)
Challenge thoughts	Control	3.0 (1.0)	3.0 (1.0)	
	Intervention	3.0 (1.0)	3.3 (1.0)	3.2 (1.1)

Problematic use of social media represents the mean of the negative consequences of social media use scale only

### Between-subjects analyses

#### Mental health

**Fig. 2:**
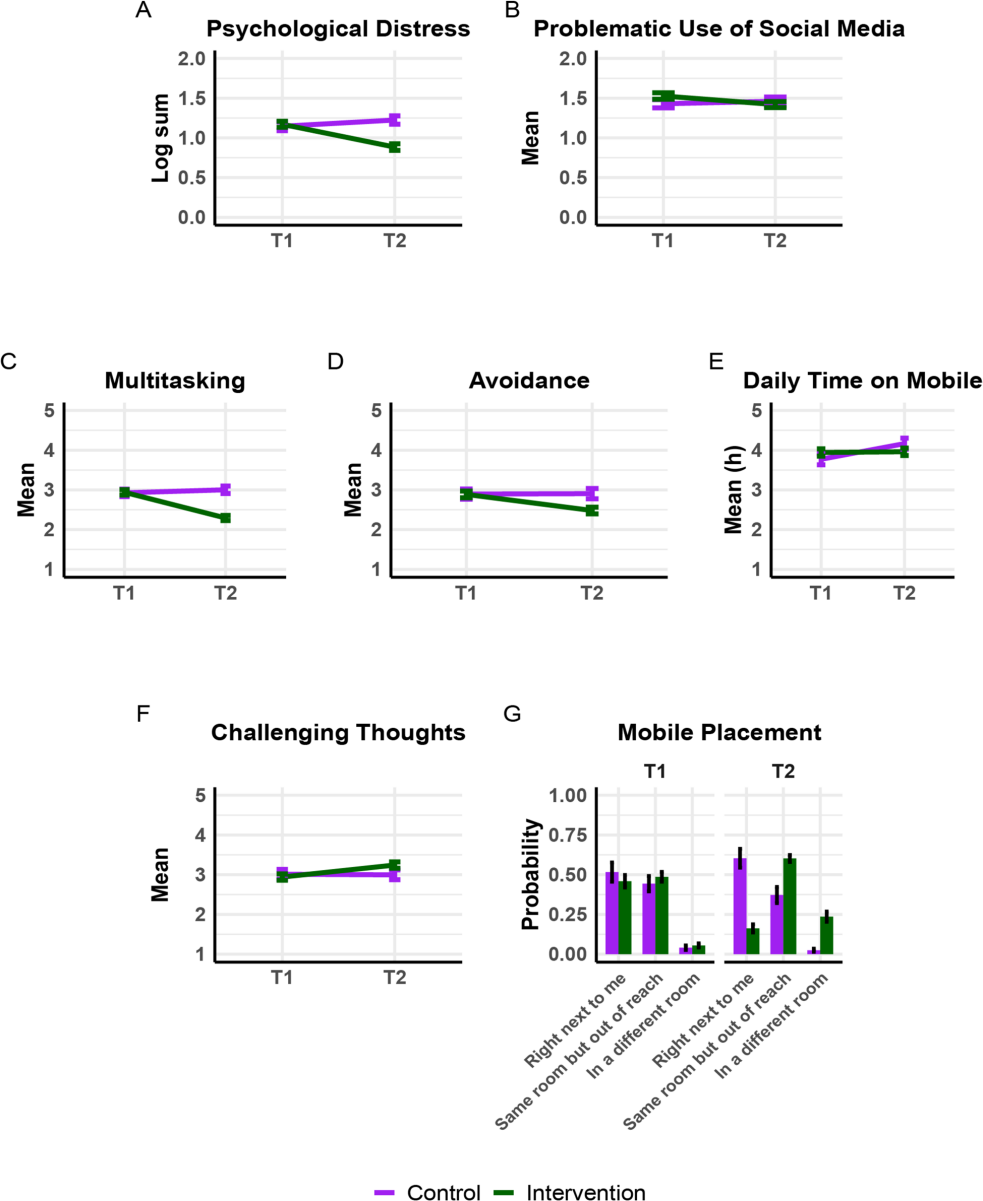
Changes in outcomes for mental health, behaviors, and strategies**.** Predicted mean values for the control and intervention groups at T1 (pre-measurement) and T2 (post-measurement) are shown for seven outcomes. Panel **A** log sum of PHQ scores (psychological distress; higher = more distress). Panel **B** GSMQ scores (problematic social media use; higher = more problems). Panel **C** multitasking while studying (higher = more multitasking). Panel **D** avoidance behaviors (higher = more avoidance). Panel **E** daily smartphone time. Panel **F** challenging negative thoughts (higher = greater engagement). Panel **G** probabilities for phone placement while studying (“right next to me,” “same room but out of reach,” or “different room”)

Three models were fitted to evaluate mental health-related outcomes. Psychological distress, measured by the PHQ-4 sum score, was modeled using a GLMM, assuming a Poisson distribution. Results indicated a significant time × group interaction (*β* = 0.69, *SE* = 0.04, *Z* = − 5.67, *p* < 0.001, R^2^_part_ = 0.012). The expected sum of psychological distress symptoms in the intervention group decreased by approximately 31% from T1 (pre-measurement) to T2 (post-measurement) compared to the control group (${e}^{-0.37}$= 0.69; Fig. 2, Panel A). As higher scores on this scale reflect greater symptoms of anxiety or depression, this change represents a positive effect of the intervention. Health-related quality of life, assessed with the KIDSCREEN-10, was modeled with a GLMM using a Poisson distribution. No significant effects of the intervention were observed on this outcome. For problematic use of social media, assessed with the GSMQ-9, the residuals were not normally distributed, and the outcome was right-skewed. Therefore, it was modeled using a GLMM with a Gamma distribution with a logit link A significant time × group interaction was found (*β* = 0.91, *SE* = 0.02, *t* = -3.89, *p* < 0.001, marginal R^2^_part_ = 0.005), indicating a reduction in problematic social media use from T1 to T2 in the intervention group relative to the control group (Fig. 2, Panel B). Higher scores on this measure reflect more problematic behaviors; thus, the observed decrease represents a beneficial effect of the intervention.

#### Behaviors

No significant intervention effects were observed for average sleep duration. Multitasking while studying was analyzed using a CLMM. Results revealed a significant time × group interaction (*β* = 0.30, *SE* = 0.23, *t* = − 5.36, *p* < 0.001, *r*^*2*^_part_ = 0.007), reflecting a reduction in multitasking for the intervention group at T2, whereas the control group remained stable between T1 and T2 (Fig. 2, Panel C). Avoidant behaviors were also analyzed with a CLMM. A significant time × group interaction was found (*β* = 0.45, *SE* = 0.23, *Z* = − 3.47, *p* < 0.001, R^2^_part_ = 0.003), indicating a decrease in avoidance in the intervention group from T1 to T2, while the control group showed no change (Fig. 2, Panel D). For negative online interactions, the distribution was right-skewed and there was an excess of zeroes. Data were therefore modelled with a zero-inflated model, with a truncated Poisson distribution, with a random intercept for gender for each participant.^1^ No significant effects of the intervention were observed for this outcome. Daily time spent with smartphone use showed a significant time × group interaction (*β* = − 0.37, *SE* = 0.13, *t*(676) =  − 2.78, *p* = 0.01, R^2^_part_ = 0.003). This effect reflected an increase in smartphone use in the control group from T1 to T2, whereas the intervention group maintained stable usage over the same period (Fig. 2, Panel E).

#### Strategies

No significant intervention effects were observed for smartphone notification settings or for the use of breathing strategies; both outcomes were modeled using CLMMs. Personal problems, assessed with the GHSQ, were analyzed using a zero-inflated model with a truncated Poisson distribution to account for excess zeroes, but no significant effects of the intervention were found. For challenging negative thoughts, a significant time × group interaction was observed (*β* = 0.32, *SE* = 0.10, *t*(696) = 3.16, *p* = 0.002, R^2^_part_ = 0.009), indicating that students in the intervention group reported higher use of this strategy at T2 compared to the control group, whose scores remained stable from T1 to T2 (Fig. 2, Panel F). Phone placement while studying was modeled with a CLMM with a probit link,^2^ and a significant time × group interaction was found (*β* = 1.10, *SE* = 0.16, *Z* = 7.05, *p* < 0.001, *r*^*2*^_part_ = 0.019). Students in the intervention group were more likely to place their smartphone further away—either elsewhere in the same room or in a different room—during study sessions at T2 compared to both their own T1 scores and the control group (Fig. 2, Panel G).

### Follow-up analyses

To examine whether intervention effects were maintained at the one-year follow-up, we analyzed a subset of participants (*n* = 122) from grade 7 for within group comparisons between T3 and T1 for the intervention group who completed all three measurement points (see Tables 30–42 in the Online Appendix for the results). The school random effect was removed since only three schools participated and there was no variation between the schools.

#### Mental health

For mental health outcomes, the only effect observed at follow-up was for psychological distress. Compared to baseline (T1), the intervention group demonstrated a lower log-transformed sum score at T3 (*β* = 0.69, *SE* = 0.05, *Z* = − 4.70, *p* < 0.001, R^2^_part_ = 0.045). The result indicates a 31% decrease in the expected sum of symptoms of depression and anxiety at T3 relative to baseline.

#### Behaviors

Three behavioral outcomes showed significant effects at follow-up. For multitasking while studying, there was a significant reduction at T3 (*β* = 0.40, *SE* = 0.25, *Z* = − 3.66, *p* < 0.001, R^2^_part_ = 0.013). The effect of time indicated that the odds of reporting higher levels of multitasking were 60% lower at T3 compared to baseline. Avoidant behaviors were also sustained at T3 (*β* = − 0.70, *SE* = 0.16, Z = − 4.81, *p* < 0.001, R^2^_part_ = 0.025) and the effect of time showed that the likelihood of reporting more avoidance behavior decreased at T3 relative to baseline. The reported daily smartphone use increased at T3 (*β* = 0.41, *SE* = 0.16, *t*(236) = 2.62, *p* = 0.01, R^2^_part_ = 0.011), to approximately the same level as the control group did one year earlier.

#### Strategies

Three strategy-related outcomes showed significant effects at follow-up. When comparing T3 to T1, students significantly more often modified their smartphone notification settings to reduce disturbances (*β* = 0.41, *SE* = 0.15, Z = 2.76, *p* = 0.006, R^2^_part_ = 0.01). They also reported significantly greater use of challenging negative thoughts (*β* = 0.25, *SE* = 0.12, *t* (238) = 2.12, *p* = 0.035, R^2^_part_ = 0.020). For phone placement while studying, students more often placed their smartphones farther away (“in the same room” or “in a different room”) at T3 compared to baseline (*β* = 0.61, *SE* = 0.17, Z = 3.64, *p* < 0.001, R^2^_part_ = 0.024), indicating sustained improvements in environmental strategies for focus and digital balance.

### Sensitivity analyses

Dose-based analyses were included as sensitivity analyses. In these analyses, the group variable (intervention versus control) was replaced with the number of modules the teacher reported completing, while keeping all other model specifications unchanged. In the intervention group, the mean number of completed modules was 4.7 (SD = 0.82), indicating that most teachers delivered all five modules. The results closely resembled those obtained using the original group variable, suggesting that variation in the number of completed modules did not meaningfully influence the observed outcomes.

### Teacher perceived benefits, satisfaction, and reports of adverse events

Finally, we examined how the teachers perceived the program. As shown in Table 3, the majority of teachers agreed (4) or “strongly agreed” (5) that the program was a valuable resource. The exception was the question regarding nervousness before presentations, for which the perceived benefits were slightly lower (M = 3.43). Teachers were also generally satisfied with the program. For example, a majority of the teachers agreed or strongly agreed with the statements that they would recommend the program to a colleague (68.4%), that the program was relevant for the students (73.7), and that the program would have long-term benefits for the students (68.4%). In open-ended questions, none of the teachers reported any adverse events when administering the program, or that any of their students appeared to have experienced negative effects due to the program.

**Table 3:** Teachers perceived program benefits and teacher satisfaction

	M (SD)	% 4 or 5
Teacher perceived benefits of the program		
The program has been a valuable resource for addressing…		
… How mobile phones can affect sleep	4.00 (0.88)	63.2
… How to deal with nervousness before a presentation	3.43 (0.65)	35.7
… How to improve students’ focus/attention	4.05 (0.71)	78.9
… How to improve students’ mental health	4.11 (0.83)	72.2
… How to achieve a better digital media balance	4.16 (0.83)	73.7
Teacher satisfaction		
I want to continue working with the program	3.83 (1.25)	55.6
I would recommend the program to a colleague	4.11 (1.15)	68.4
I think that the program is relevant for the students	4.16 (0.83)	73.7
I notice that the students connect with the messages in the program	3.37 (0.96)	47.4
I think that the program will have long-term benefits for the students	4.00 (0.82)	68.4

## Discussion

This study evaluated the effects of a school-based program combining digital media balance and SEL, designed to target three key areas critical for adolescent mental and physical health. We hypothesized that participation in the program would lead to improvements in mental health, greater adoption of adaptive behaviors, and increased use of strategies introduced during the intervention. Mental health outcomes were assessed using three validated measures assessing psychological distress, health-related quality of life, and problematic use of social media. Target behaviors included sleep, multitasking, avoidance, negative online interactions, and daily smartphone use, while strategies encompassed approaches to promote positive behaviors and reduce negative ones. Overall, the findings indicate that the intervention had beneficial effects across mental health, behavioral, and strategy-related domains, and some effects were maintained at the one-year follow-up.

### Hypothesis I: Enhanced outcomes of mental health

The first hypothesis proposed that adolescents in the intervention group would exhibit more favorable mental health outcomes compared to the control group, driven by the cumulative impact of the program’s five modules. In line with this expectation, a small but significant improvement in psychological distress was observed in the intervention group at the second measurement point relative to the control group. Notably, this effect was also evident at the third measurement point, indicating a lasting benefit up to 12 months post-intervention. These findings are consistent with prior reviews of SEL programs, which have also reported improvements in emotional distress and related outcomes [46]. No significant intervention effect was detected for health-related quality of life. This may be partly explained by the broad scope of such measures, assessing factors ranging from energy levels and school performance to perceptions of parental fairness—areas not targeted by the intervention. This result is consistent with findings from a meta-analysis of a range of well-being programs, which reported limited effects on general well-being [43]. One contributing factor may be the stronger associations observed between social media use and mental health *problems*, rather than overall well-being [75]. In line with this, the intervention had a significant effect in reducing problematic use of social media, reinforcing previous conclusions from other school-based interventions [52]. Adolescents in the intervention group reported fewer negative consequences of social media than the control group and their own baseline reports. Given the previously mentioned links between problematic use of social media and mental health problems [21, 22, 27, 75], this is an important result. This reduction likely also contributed to improvements in psychological distress, even if broader quality-of -life measures remained unchanged. However, the effect on social media-related negative consequences was not sustained 1-year post-intervention, highlighting the need for sustained efforts to maintain these benefits over time.

### Hypothesis II: Improved health behaviors

The second hypothesis proposed that the intervention would reduce unhealthy behaviors associated with dysfunctional digital media use, such as displacing sleep [15], using media to escape negative emotions [25], or multitasking with media while studying [35]. The study did not detect an intervention effect on sleep duration, which may reflect the need for additional caregiver support as previously suggested by Jakobsson et al. [6], or more intensive intervention components to influence sleep routines. A previous school-based intervention specifically targeting sleep quality found effects on attitudes and beliefs about sleep, but not actual sleep behaviors [76], further suggesting that sleep routines may be more resistant to change with merely psychoeducational efforts.

A significant effect of the intervention was observed for daily smartphone use. Students in the intervention group maintained consistent use throughout the study, whereas the control group displayed an increase, consistent with prior reports showing rising digital media use with age [10, 11, 77]. Although maintaining baseline screen time may be considered a positive outcome, these results highlight that reducing usage may be more challenging and may require more structural strategies, involving changes to habits, lifestyle, and social norms. Given well-documented associations between screen time and sleep disruption [15, 17, 78] and the benefits of reducing social media use for emotionally distressed individuals [33], continued exploration of interventions that address both problematic digital media use and reduce overall screen time is warranted.

The intervention also targeted dysfunctional use through emotion regulation and exposure-based strategies. Drawing on the Digital Media-use Effects in children and adolescents model [34], our intervention program emphasized understanding the affective states driving digital media use (e.g., stress, boredom) and the contextual factors surrounding it (e.g., avoidance, smartphone placement). A module addressing avoidant behaviors incorporated basic cognitive-behavioral principles to practice frustration tolerance and resilience in the face of discomfort. Results indicated a small but lasting reduction in avoidance, supporting the effectiveness of cognitive-behavioral strategies in school-based interventions, consistent with prior research on preventing internet and gaming addiction [52].

Regarding multitasking with digital media during homework, the intervention group showed a reduction compared with the control group, and this effect persisted one year later. Previous interventions targeting multitasking among adults have shown modest effects using technical restrictions (e.g., blockers or timers), limited effects from awareness alone, and some effects via mindfulness exercises [79, 80]. To our knowledge, this study is the first to report effects on adolescent media multitasking in a school-based program. Although we did not test mediating effects in this study, media multitasking has previously been linked to avoidance of negative emotions [81]. Thus, it is possible that the effects on multitasking have been reinforced by improvements in emotion regulation, as evidenced by reductions in avoidance and psychological distress. However, this is for future studies to discern. Regardless, these outcomes are relevant given that multitasking can impede academic performance [35, 36] and cognitive development [38, 39]. Thus, sustained improvements could have long-term cumulative benefits. No intervention effects were observed for negative online interactions occurring in the past few weeks, likely due to a floor effect, as most participants reported zero events at baseline, leaving little room for improvement.

### Hypothesis III: Increased strategy utilization

The intervention introduced several strategies across its modules to support healthy habits, emotion regulation, and balanced digital media use. It was therefore hypothesized that the intervention group would demonstrate greater utilization of these strategies after the intervention compared to the control group.

One strategy promoted in the intervention was modifying smartphone settings to create additional friction, such as disabling notifications, rearranging apps on the home screen, setting time limits, and switching the display to grayscale. While a previous intervention promoting ten such strategies showed reductions in screen time and problematic smartphone use [59], more recent interventions suggest that the effects may be tied more specifically to switching to grayscale [82], or installing blocking apps [83], rather than disabling notifications [84]. In the current study, the only strategy assessed was disabling notifications, which was not influenced by the intervention. This illustrates the need for school-based programs to continuously stay updated on developments in the research field, as well as the importance of selecting relevant outcomes. A second strategy addressed in the intervention involved challenging negative thoughts, where students practiced cognitive reappraisals and cognitive distortion management. The intervention group showed significant improvements in this strategy, which persisted at follow-up, suggesting stable change. This skill likely contributed to reductions in psychological distress and avoidance behaviors, as challenging negative thoughts is a core component of cognitive-behavioral therapy and standard anxiety treatment [85]. Supporting this, Throuvala et al. [86] found that improved self-awareness mediated the effect of a digital media intervention on smartphone distraction, highlighting the importance of combining SEL and cognitive strategies with environmental adjustments for balanced digital media use [34].

A third strategy targeted phone placement during study sessions. Results showed that students in the intervention group were more likely to place their smartphones out of reach or in a different room, rather than keeping the phones next to them, compared with the control group. Minimizing proximity to smartphones has been shown to reduce media multitasking and improve cognitive capacity during academic activities [35, 36, 40]. By changing the immediate environment, this strategy may have contributed to the observed decline in multitasking behaviors. To our knowledge, no previous studies have examined this strategy in adolescents.

The fourth strategy involved breathing techniques, which were briefly introduced in the intervention with exercises such as box breathing. However, no group differences were detected. Mindfulness-based breathing has been shown to enhance psychological well-being and help to manage anxiety [87], and previous school-based interventions have reported positive effects in 11–13-year-olds, although caution is advised for younger children [45].

Finally, the intervention encouraged communication about personal and emotional problems with peers, parents, and school staff. No significant effects were observed, which may reflect a reduced need for such communication due to improvements in psychological distress. Similarly, other school-based and web-based interventions have reported limited effects on help-seeking despite positive outcomes on other measures [44, 88].

### Sensitivity analysis

Sensitivity analysis incorporated the number of completed modules into regression models to control for dose effects. Results remained qualitatively consistent, suggesting that the observed effects were not dose dependent. However, this may be explained by the program’s high completion rate (an average of 4.7 modules out of 5). Thus, the method of using a video-based program delivering scientifically based facts, with discussions and exercises facilitated by teachers or healthcare personnel with limited preparation, demonstrated high feasibility.

### Clinical relevance

Regarding the strengths of the effects, small, standardized effects (i.e., partial R^2^ = 0.003–0.012; [97], p. 41) can still be meaningful when the intervention is low‐cost, school-wide, and scalable. In addition, our effects cross empirically and clinically meaningful thresholds on their respective measures. For example, the PHQ-4 sum score fell from 3.5 to 2.7 from T1 to T2, a 31% reduction. On the PHQ-4, scores of 3–5 denote “mild” distress and 0–2 “minimal” distress [89]. Thus, our participants shifted from the lower end of the mild range into the minimal range, which meant they crossed the very cut‐point validated in a general population [90]. Regarding the 0.2-point drop on the GSMQ-9, which represents an 11% relative reduction in problematic social-media behaviors, we refer to Fassi et al. [91]. They showed that a one-point increase on similar social-media measures corresponds to cross-sectional *r* = 0.12 with internalizing symptoms in clinical adolescent samples. By that logic, our 0.2-point decrease should lower expected mental-health risk by approximately 20% of that *r* = 0.12 increment, that is, a *r* = 0.02 shift.

### Strengths, limitations and future directions

Reversing the trend of increasing mental health concerns in adolescents requires coordinated efforts across schools, families, and communities. Universal school-based programs can reach all students, including those who are disadvantaged or less likely to seek healthcare services or information about digital media balance.

Regarding strengths, the intervention was feasible to deliver; most teachers completed all five modules with minimal time and resources, preceded by an extensive development phase. This suggests that the program can be administered at a low cost and is scalable. In general, teachers also perceived the program as being relevant and potentially having long-term benefits. Teachers were also generally satisfied with the program, and a majority would recommend it to a colleague. No adverse events were reported. The program encouraged student engagement through dramatized videos featuring third-party characters, a method that effectively reduced stigma and increased relatability [61]. Utilizing teachers rather than external facilitators enabled ongoing discussions and adaptations, aligning with research on teacher-student relationships in SEL programs [46]. Furthermore, the intervention met the SAFE criteria for effective SEL programs [49] by using sequenced learning and skill practice first applied to fictional characters and then to personal experiences. Thus, in summary, the program should be considered feasible. While effects were generally small, it has been argued that these types of effects could have meaningful, cumulative effects over time [92].

Although the study findings are promising, there are some limitations and avenues for future studies worth mentioning. The study's non-randomized design may have introduced expectancy effects; however, such biases likely did not affect students, as the program was introduced as part of the standard curriculum. Furthermore, using a convenience sample, in which the participating intervention schools were already engaged in the school program, may have facilitated a more successful implementation, than would be expected in schools recruited specifically for the study. However, time, engagement, and endorsement from leadership and other key stakeholders are essential to most successful school interventions [93, 94] and should perhaps be regarded as a prerequisite for investigating effects. Using multilevel modeling, we could accommodate different cluster sizes and nonrandom assignment by simultaneously partialling out between‐ and within‐school variance [95]. Baseline covariates (SES, age, gender) were included in the model to further protect against residual confounding due to baseline imbalance and well-documented gender differences [66]. Even though we included gender as a covariate, we did not investigate gender differences in program effectiveness, which will be an important avenue for future research.

Further, self-report measures include inherent limitations related to objectivity and social desirability biases. Future studies should consider more objective measures, such as attendance records or healthcare utilization. In addition, only the younger age group was followed up due to final exams in the older group, limiting the sample size and power to detect sustained effects.. Furthermore, the control group could not be followed at the third measurement point because they had implemented the program by then. As a result, long-term effects cannot be directly compared to controls, limiting conclusions about sustained impact.

While the intervention reduced problematic social media use, it did not decrease overall smartphone time or improve sleep behaviors. The persistence of high smartphone use and insufficient sleep reflects how common and normalized these behaviors have become. Such entrenched patterns point to broader cultural and environmental factors that may limit what can be achieved through school-based interventions alone. In this study, 63% of the teachers sent out the parent-facing videos, implying that not all components of the program were fully implemented. Coordinated efforts to engage caregivers and policy measures, such as phone-use restrictions or campaigns shifting social norms, are needed. Without such structural changes, school-based interventions may continue to face the challenge of prevailing norms. Further, educator professional development that aligns SEL competencies with cultural values is crucial. Finally, identifying the conditions under which behavioral change is most likely to occur will be key to improving the effectiveness of universal school-based programs targeting mental health and digital media balance.

## Conclusions

The evaluation of the SEL and digital balance program “On the Inside” indicates promising effects on students’ mental health outcomes, adaptive behaviors, and strategic use of skills to manage the digital environment. The intervention demonstrated positive effects across several domains, including reductions in problematic social media use and psychological distress. These changes may have been driven by behavioral strategies, such as reducing multitasking, placing phones out of reach while studying, challenging unhelpful thoughts, and avoiding difficulties less. Notably, some effects were also evident one year after the intervention, highlighting potential for lasting impact.

The effect sizes were small. However, this aligns with previous mental health prevention programs [96]. Importantly, even modest individual-level effects can translate into meaningful outcomes when scaled across large populations [97–99]. For example, Funder and Ozer [92] highlight that ibuprofen’s effect on headaches—though small—is highly relevant because headaches are so common. As long as the proportion of youth seeking health care services increases, interventions with small effects may also be cost-efficient and relevant. In summary, given the growing number of young people seeking mental health support and struggling to maintain balanced digital media use, universal school-based programs may offer meaningful benefits at a population level.

## Supplementary Information

Supplementary Material 1.

## Data Availability

The datasets used and/or analyzed during the current study are available from the corresponding author on reasonable request.

## Abbreviations

SEL: Social and emotional learning
GLMM: Generalized linear mixed model
CLMM: Cumulative link mixed models
PHQ: Patient health questionnaire
GSMQ-9: Gaming and social media questionnaire
GHSQ: General help-seeking questionnaire
SAFE criteria targeting specific SEL skills: Sequenced, Active, Focused and Explicit
ADHD: Attention-Deficit/Hyperactivity Disorder
ASD: Autism spectrum disorder

## Footnotes

1 In the conditional model, random effects were specified for the intercept of the variable “gender” within each subject, allowing for individual variation in the effects of gender on the outcome variable. In this way, we captured the student-specific differences that may impact the relationship between gender and the “negative online interactions”.

2 When fitting “phone location when studying” we used the probit link, rather than the logit link, since it provided a much better fit to data. In short, the coefficients generated by the probit link are on a Z-score scale. Hence, one unit change in X is associated with a one unit change in the Z-score of Y. To make the coefficients more interpretable they are often transformed to predicted probabilities.
