# Supplementary material for: Digital media balance and mental health: effects of a school-based program
Source: Child Adolesc Psychiatry Ment Health. 2026 Jul 10;20:95. doi: 10.1186/s13034-026-01131-3 (PMC13383498; doi:10.1186/s13034-026-01131-3)
Supplement: Supplementary file 1 — Supplementary Material 1. [file 13034_2026_1131_MOESM1_ESM.docx]

**Appendix**

**Table 1**

*Description of the intervention “On the inside” in more detail, and which SEL-Components are represented using the CASEL-Framework.*

| Module | Scientific base and expert profile | Main message | Exercise | SEL-domain |
| --- | --- | --- | --- | --- |
| Healthy mind platter | Protective factors of mental health. Healthy mind platter  - Medical doctor  - Researcher | We can deal with difficult thoughts and feelings better when we meet our brains basic needs. Module explains the science behind the factors in the Healthy mind platter. Highlights sleep, exercise and relationships. | Reflection on character struggling with mood, stress and school work and his daily habits. Design a new schedule for the character considering the basic needs. Using a work sheet to map their own basic needs. | Self management  Responsible decision making  Social awareness  Relationship skills |
| Perform without anxiety | Cognitive behavioral therapy (e.g., avoidance, anxiety curve, exposure effects and acceptance commitment therapy strategies)  - Psychologist  - Researcher | Our brains are wired to protect us from possible threats and putting ourselves at risk for being excluded from the group is one of our greatest fears. There are steps we can take to manage those fears and get the job done. By performing even though we are nervous, we are retraining our brains and that situation becomes less scary over time. | Reflection on characters' difficult situations and what might be happening in their bodies and thoughts. Use worksheet to help them prepare for the situation in advance by practicing and focusing on outcomes they can control while avoiding avoidance. Test breathing strategies, calming thought techniques and discuss how others can be supportive. | Self awareness  Self management  Responsible decision making  Social awareness |
| Take control of your attention | Cognitive functions and learning, attention, working memory, long term memory and the effect of task switching   - Researcher | Our attention is a limited resource that is easily distracted. When we are trying to learn, we can be 4 x as efficient if we focus on one thing at a time, rather than multitask. Placing your phone further away frees up attentional resources to get the job done faster and better. | Reflection on character who is media multitasking and being distracted by digital media while studying. Task-switching experiment to experience the decline in productivity while multitasking. Make a plan for single-tasking (e.g., remove phone from study area). | Self awareness  Self management  Responsible decision making |
| Shape your brain | Thought distortions, cognitive reappraisals and gender roles in media.   - Marketing specialist (female) - Author on toxic masculinity (male) - Researcher | The brain is plastic and will learn automatic thought patterns with repeated exposure. We can learn to identify overly negative thought patterns that hold us back and challenge them. It matters what we feed our brains with why it is important to critically reflect on social media messages and be careful with our digital diet. | Reflection on characters thought distortion with each other and within themselves. Practice identifying thought distortions and challenging them with alternative thoughts (cognitive reappraisals). Reflecting on media messaging and curative social media. | Self awareness  Self management  Responsible decision making  Social awareness  Relationship skills |
| Social media and digital gaming | Interaction design in apps and their influence our digital behaviors  - Professional gamer  - Music artist/  Influencer  - Researcher | Social media and digital games have been designed to keep us hooked for as long and as often as possible using specific mechanisms. Learning to identify these features can help us take control over our time. Utilized in our favour, these mechanisms can help us design the behaviors we would like to do more of. | Reflections on time spent on digital media and  behavioral design choices in apps and our lives. Which mechanisms can be applied to gain control over digital media use. Make a plan to gain control over digital media use with strategies and settings on the phone. | Self awareness  Self management  Responsible decision making  Social awareness |

**Table 2**

*Measures Used Before and After the Intervention*

| Outcome domain | Outcome variable | Scale | Predictors and covariates |
| --- | --- | --- | --- |
| Mental health related | - Psychological distress (PHQ-4) - Health related quality of life (KIDSCREEN-10) - Problematic use of social media (subset of GSMQ-9) | Interval scale, 0-3, sum score (0-12)  Sum score  Likert, 0-4, mean score | Time (pre-post test), gender (boy/girl/other), SES, intervention group (control/intervention) or dose of intervention (0-5 modules) |
| Behaviors | - Sleep - Multitasking - Avoidance - Problematic online interactions - Time on smartphone | Continuous, h/night  Likert, 1-5  Likert, 1-5  Continuous, events/month  (sum score)  Continuous, h/day | Time (pre-post test), gender, SES, intervention group (control/intervention)  Time (pre-post test), gender, SES, intervention group (control/intervention) |
| Strategies | Smartphone notifications  Breathing techniques  Talking about problems, (GHSQ)  Challenging thought distortions  Phone placement during studying | Likert, 1-5  Likert, 1-5  Sum score  Likert, 1-5  Categorical, 1-3 |  |

*Note*. For each outcome domain there are three model sets, one for between-subjects, one for the dose-based analyses, and one for
the follow-up (within-subjects) analyses.

**Table 3**

*Psychological distress (PHQ sum) multilevel between-subjects model with poisson distribution*

| Coefficient | Estimate | *SE* | *Z* | *p-*value |
| --- | --- | --- | --- | --- |
| Intercept | 2.49 | 0.19 | 11.84 | <.001 |
| Group (Intervention) | 1.03 | 0.06 | 0.4 | 0.69 |
| Time (T2) | 1.08 | 0.06 | 1.47 | 0.14 |
| Gender (Girl) | 1.48 | 0.07 | 8.58 | <.001 |
| Housing (Rental) | 0.92 | 0.07 | -1.06 | 0.29 |
| Housing (Villa) | 0.94 | 0.06 | -1.00 | 0.32 |
| Grade (8) | 1.18 | 0.06 | 3.22 | <.001 |
| Group: Time | 0.69 | 0.04 | -5.67 | <.001* |
| ICC idcode:school | 0.97 |  |  |  |
| ICC school | 0.03 |  |  |  |
| Number of observations | 1434 |  |  |  |
| AIC | 6098.48 |  |  |  |
| Model R2 | 0.125 |  |  |  |
| Interaction R2 (group:time) | 0.0119 |  |  |  |

*Note*. The random effects were students nested in schools. Baseline for categorical variables: Time: T1; Group: control; Gender: Boy; Housing: Owned apartment, Grade: 7. Estimates are exponentiated.
*Interaction remains significant after conducting a Benjamin-Hochberg FDR correction across the 13 interaction tests (adjusted *p* < .05., smallest adjusted *p* = .0003).

**Table 4**

*Quality of Life (KIDSSCREEN 10) Multilevel Between Subjects Model with Poisson Distribution*

| Effect | Estimate | *SE* | *Z* | *p*-value |
| --- | --- | --- | --- | --- |
| Intercept | 35.23 | 0.62 | 202.76 | <.001 |
| Group (Intervention) | 1.01 | 0.02 | 0.56 | 0.58 |
| Time (T2) | 1.01 | 0.02 | 0.61 | 0.54 |
| Gender (Girl) | 0.98 | 0.01 | -2.02 | 0.04 |
| Housing (Rental) | 1.02 | 0.02 | 1.02 | 0.31 |
| Housing (Villa) | 1.03 | 0.01 | 1.79 | 0.07 |
| Grade (8) | 0.97 | 0.01 | -2.56 | 0.01 |
| Group: Time | 0.98 | 0.02 | -0.82 | 0.41 |
| ICC idcode:school | 2.5e-13 |  |  |  |
| ICC school | 1.00 |  |  |  |
| Number of observations | 1401 |  |  |  |
| AIC | 8260.35 |  |  |  |
| Model R2 | 0.0103 |  |  |  |
| Interaction R2 (group:time) | 0.0005 |  |  |  |

*Note*. The random effects were students nested in schools. Baseline for categorical variables: Time: T1; Group: control; Gender: Boy; Housing: Owned apartment, Grade: 7. Estimates are exponentiated.

**Table 5**

*Negative Consequences (GSMQ) Multilevel  Between Subjects with Gamma Distribution*

| Effect | Estimate | *SE* | *Z* | *p*-value |
| --- | --- | --- | --- | --- |
| Intercept | 1.60 | 0.06 | 4.82 | <.001 |
| Group (Intervention) | 1.07 | 0.05 | 1.49 | 0.14 |
| Time (T2) | 1.02 | 0.02 | 1.05 | 0.29 |
| Gender (Girl) | 1.24 | 0.04 | 6.94 | <.001 |
| Housing (Rental) | 0.98 | 0.04 | -0.41 | 0.68 |
| Housing (Villa) | 1.01 | 0.03 | 0.4 | 0.69 |
| Grade (8) | 1.04 | 0.04 | 1.12 | 0.26 |
| Group: Time | 0.91 | 0.02 | -3.89 | <.001* |
| ICC idcode: school | 0.57 |  |  |  |
| ICC school | 0.00 |  |  |  |
| Number of observations | 1352 |  |  |  |
| AIC | 1374.21 |  |  |  |
| Model R2 | 0.0838 |  |  |  |
| Interaction R2 (group:time) | 0.0046 |  |  |  |

*Note*. The random effects were students nested in schools. Baseline for categorical variables: Time: T1; Group: control; Gender: Boy; Housing: Owned apartment, Grade: 7. Estimates are exponentiated.
*Interaction remains significant after conducting a Benjamin-Hochberg FDR correction across the 13 interaction tests (adjusted *p* < .05., smallest adjusted *p* = .0003).

**Table 6**

*Average Sleep Multilevel Cumulative Between Subjects Model with a Log Link*

| Effect | Estimate | *SE* | *Z* | *p*-value |
| --- | --- | --- | --- | --- |
| 6\|7 | 0.01 | 0.4 | -10.61 | <.001 |
| 7\|8 | 0.17 | 0.37 | -4.76 | <.001 |
| 8\|9 | 2.89 | 0.37 | 2.88 | <.001 |
| 9\|10 | 43.57 | 0.4 | 9.39 | <.001 |
| Group (Intervention) | 1.04 | 0.35 | 0.1 | 0.92 |
| Time (T2) | 0.74 | 0.2 | -1.47 | 0.14 |
| Gender (Girl) | 0.64 | 0.17 | -2.68 | 0.01 |
| Housing (Rental) | 0.80 | 0.29 | -0.79 | 0.43 |
| Housing (Villa) | 0.90 | 0.25 | -0.42 | 0.68 |
| Grade (8) | 0.39 | 0.24 | -3.87 | <.001 |
| Group: Time | 1.09 | 0.24 | 0.36 | 0.72 |
| ICC idcode:school | 0.50 |  |  |  |
| ICC school | 0.04 |  |  |  |
| Number of observations | 1454 |  |  |  |
| AIC | 3880.04 |  |  |  |
| Model R2 | 0.0123 |  |  |  |
| Interaction R2 (group:time) | 0.00 |  |  |  |

*Note*. The random effects were students nested in schools. Baseline for categorical variables: Time: T1; Group: control; Gender: Boy; Housing: Owned apartment, Grade: 7. Estimates are exponentiated.

**Table 7**

*Multitasking while Studying Cumulative Between Subjects Model with a Log Link*

| Effect | Estimate | *SE* | *Z* | *p*-value |
| --- | --- | --- | --- | --- |
| 1\|2 | 0.20 | 0.23 | -6.96 | <.001 |
| 2\|3 | 0.82 | 0.22 | -0.9 | 0.37 |
| 3\|4 | 3.63 | 0.23 | 5.7 | <.001 |
| 4\|5 | 18.67 | 0.25 | 11.75 | <.001 |
| Group (Intervention) | 1.02 | 0.18 | 0.11 | 0.91 |
| Time (T2) | 1.13 | 0.19 | 0.67 | 0.5 |
| Gender (Girl) | 1.87 | 0.12 | 5.05 | <.001 |
| Housing (Rental) | 1.21 | 0.22 | 0.88 | 0.38 |
| Housing (Villa) | 1.12 | 0.18 | 0.63 | 0.53 |
| Grade (8) | 1.09 | 0.14 | 0.63 | 0.53 |
| Group: Time | 0.30 | 0.23 | -5.36 | <.001* |
| ICC idcode:school | 0.18 |  |  |  |
| ICC school | 0.01 |  |  |  |
| Number of observations | 1406 |  |  |  |
| AIC | 4244.97 |  |  |  |
| Model R2 | 0.0331 |  |  |  |
| Interaction R2 (group:time) | 0.0069 |  |  |  |

*Note*. The random effects were students nested in schools. Baseline for categorical variables: Time: T1; Group: control; Gender: Boy; Housing: Owned apartment, Grade: 7. Estimates are exponentiated.
*Interaction remains significant after conducting a Benjamin-Hochberg FDR correction across the 13 interaction tests (adjusted *p* < .05., smallest adjusted *p* = .0003).

**Table 8**

*Avoidance Between Subjects Cumulative Multilevel Model with a Log Link*

| Effect | Estimate | *SE* | *Z* | *p*-value |
| --- | --- | --- | --- | --- |
| 1\|2 | 0.21 | 0.31 | -4.98 | <.001 |
| 2\|3 | 1.30 | 0.31 | 0.85 | 0.39 |
| 3\|4 | 7.27 | 0.32 | 6.28 | <.001 |
| 4\|5 | 40.31 | 0.34 | 10.86 | <.001 |
| Group (Intervention) | 0.98 | 0.28 | -0.05 | 0.96 |
| Time (T2) | 1.02 | 0.19 | 0.11 | 0.91 |
| Gender (Girl) | 4.65 | 0.16 | 9.81 | <.001 |
| Housing (Rental) | 0.83 | 0.25 | -0.71 | 0.48 |
| Housing (Villa) | 0.93 | 0.22 | -0.32 | 0.75 |
| Grade (8) | 1.57 | 0.21 | 2.14 | 0.03 |
| Group: Time | 0.45 | 0.23 | -3.47 | <.001* |
| ICC idcode:school | 0.43 |  |  |  |
| ICC school | 0.01 |  |  |  |
| Number of observations | 1438 |  |  |  |
| AIC | 4238.79 |  |  |  |
| Model R2 | 0.0388 |  |  |  |
| Interaction R2 (group:time) | 0.0029 |  |  |  |

*Note*. The random effects were students nested in schools. Baseline for categorical variables: Time: T1; Group: control; Gender: Boy; Housing: Owned apartment, Grade: 7. Estimates are exponentiated.
*Interaction remains significant after conducting a Benjamin-Hochberg FDR correction across the 13 interaction tests (adjusted *p* < .05., smallest adjusted *p* = .002).

**Table 9**

*Negative Online Interactions Zero-Inflated Multilevel Between Subjects Model with Truncated Poisson distribution*

| Component | Effect | Estimate | *SE* | *Z* | *p-value* |
| --- | --- | --- | --- | --- | --- |
| Conditional model | Intercept | 2.68 | 0.8 | 3.31 | <.001 |
| Conditional model | Group (Intervention) | 0.92 | 0.24 | -0.34 | 0.73 |
| Conditional model | Time (T2) | 0.97 | 0.13 | -0.22 | 0.82 |
| Conditional model | Gender (Girl) | 0.66 | 0.16 | -1.77 | 0.08 |
| Conditional model | Housing (Rental) | 1.50 | 0.43 | 1.39 | 0.16 |
| Conditional model | Housing (Villa) | 0.61 | 0.16 | -1.91 | 0.06 |
| Conditional model | Grade (8) | 1.44 | 0.32 | 1.61 | 0.11 |
| Conditional model | Group: Time | 0.78 | 0.14 | -1.35 | 0.18 |
| Zero model | Intercept | 2.66 | 0.62 | 4.18 | <.001 |
| Zero model | Group (Intervention) | 0.94 | 0.2 | -0.29 | 0.77 |
| Zero model | Time (T2) | 1.14 | 0.29 | 0.52 | 0.6 |
| Zero model | Housing (Rental) | 1.10 | 0.25 | 0.42 | 0.68 |
| Zero model | Housing (Villa) | 1.53 | 0.3 | 2.16 | 0.03 |
| Zero model | Grade (8) | 0.88 | 0.13 | -0.86 | 0.39 |
| Zero model | Group: Time | 1.28 | 0.38 | 0.83 | 0.4 |
|  | ICC idcode (Intercept) | 0.75 |  |  |  |
|  | Number of observations | 1400 |  |  |  |
|  | AIC | 2784.61 |  |  |  |
|  | Model R2 | 0.1026 |  |  |  |
|  | Interaction R2 (group:time) | -0.0025 |  |  |  |

*Note*. The model includes random intercepts and slopes for the effect of sex, which are nested within students (idcode) due to the lack of variation of students nested within schools. Baseline for categorical variables: Time: T1; Group: control; Gender: Boy; Housing: Owned apartment, Grade: 7. Estimates are exponentiated.

**Table 10**

*Daily Time on Mobile Multilevel Between Subjects Linear Model*

| Effect | Estimate | *SE* | *t* | *df* | *p-*value |
| --- | --- | --- | --- | --- | --- |
| Intercept | 3.33 | 0.18 | 18.3 | 12.92 | <.001 |
| Group (Intervention) | 0.17 | 0.16 | 1.06 | 4.76 | 0.34 |
| Time (T2) | 0.39 | 0.11 | 3.42 | 663.39 | <.001 |
| Gender (Girl) | 0.63 | 0.10 | 6.5 | 847.77 | <.001 |
| Housing (Rental) | -0.13 | 0.16 | -0.81 | 1004.59 | 0.42 |
| Housing (Villa) | -0.09 | 0.14 | -0.63 | 282.35 | 0.53 |
| Grade (8) | 0.41 | 0.12 | 3.3 | 12.68 | 0.01 |
| Group: Time | -0.37 | 0.13 | -2.78 | 676.58 | 0.01* |
| ICC idcode:school | 0.51 |  |  |  |  |
| ICC school | 0.01 |  |  |  |  |
| Number of observations | 1416 |  |  |  |  |
| AIC | 5116.11 |  |  |  |  |
| Model R2 | 0.0572 |  |  |  |  |
| Interaction R2 (group:time) | 0.003 |  |  |  |  |

*Note*. The random effects were students nested in schools. Baseline for categorical variables: Time: T1; Group: control; Gender: Boy; Housing: Owned apartment, Grade: 7.
*Interaction remains significant after conducting a Benjamin-Hochberg FDR correction across the 13 interaction tests (adjusted *p* < .05., smallest adjusted *p* = .011).

**Table 11**

*Notifications Settings Cumulative Multilevel Between Subjects Model with a Log Link*

| Effect | Estimate | *SE* | *Z* | *p*-value |
| --- | --- | --- | --- | --- |
| 1\|2 | 0.16 | 0.26 | -7.1 | <.001 |
| 2\|3 | 0.33 | 0.26 | -4.37 | <.001 |
| 3\|4 | 1.42 | 0.25 | 1.41 | 0.16 |
| 4\|5 | 6.02 | 0.26 | 6.9 | <.001 |
| Group (Intervention) | 0.76 | 0.2 | -1.34 | 0.18 |
| Time (T2) | 1.09 | 0.2 | 0.43 | 0.66 |
| Gender (Girl) | 1.44 | 0.14 | 2.61 | 0.01 |
| Housing (Rental) | 0.58 | 0.24 | -2.23 | 0.03 |
| Housing (Villa) | 1.00 | 0.2 | 0.004 | 1 |
| Grade (8) | 0.88 | 0.16 | -0.79 | 0.43 |
| Group: Time | 1.06 | 0.23 | 0.26 | 0.8 |
| ICC idcode:school | 0.36 |  |  |  |
| ICC school | 0.00 |  |  |  |
| Number of observations | 1408 |  |  |  |
| AIC | 4356.56 |  |  |  |
| Model R2 | 0.0095 |  |  |  |
| Interaction R2 (group:time) | 0.00 |  |  |  |

*Note*. The random effects were students nested in schools. Baseline for categorical variables: Time: T1; Group: control; Gender: Boy; Housing: Owned apartment, Grade: 7. Estimates are exponentiated.

**Table 12**

*Breathing Strategies Cumulative Multilevel Between Subjects Model with a Log Link*

| Effect | Estimate | *SE* | *Z* | *p-*value |
| --- | --- | --- | --- | --- |
| 1\|2 | 0.26 | 0.33 | -4.05 | <.001 |
| 2\|3 | 0.99 | 0.33 | -0.04 | 0.97 |
| 3\|4 | 5.67 | 0.33 | 5.23 | <.001 |
| 4\|5 | 35.42 | 0.35 | 10.12 | <.001 |
| Group (Intervention) | 0.97 | 0.31 | -0.11 | 0.91 |
| Time (T2) | 1.01 | 0.19 | 0.04 | 0.96 |
| Gender (Girl) | 1.37 | 0.14 | 2.22 | 0.03 |
| Housing (Rental) | 0.96 | 0.25 | -0.18 | 0.86 |
| Housing (Villa) | 1.30 | 0.22 | 1.2 | 0.23 |
| Grade (8) | 0.82 | 0.21 | -0.98 | 0.33 |
| Group: Time | 1.51 | 0.23 | 1.82 | 0.07 |
| ICC idcode:school | 0.35 |  |  |  |
| ICC school | 0.03 |  |  |  |
| Number of observations | 1440 |  |  |  |
| AIC | 4359.56 |  |  |  |
| Model R2 | 0.0103 |  |  |  |
| Interaction R2 (group:time) | 0.0008 |  |  |  |

*Note*. The random effects were students nested in schools. Baseline for categorical variables: Time: T1; Group: control; Gender: Boy; Housing: Owned apartment, Grade: 7. Estimates are exponentiated.

**Table 13**

*Talk about Problems (GHSQ) Zero-Inflated Multilevel Between Subjects Model with Truncated Poisson Link*

| Component | Effect | Estimate | *SE* | *Z* | *p-*value |
| --- | --- | --- | --- | --- | --- |
| Conditional model | Intercept | 1.82 | 0.15 | 7.46 | <.001 |
| Conditional model | Group (Intervention) | 1.10 | 0.07 | 1.57 | 0.12 |
| Conditional model | Time (T2) | 0.99 | 0.06 | -0.14 | 0.89 |
| Conditional model | Gender (Girl) | 1.07 | 0.05 | 1.43 | 0.15 |
| Conditional model | Housing (Rental) | 0.98 | 0.08 | -0.29 | 0.77 |
| Conditional model | Housing (Villa) | 0.91 | 0.06 | -1.37 | 0.17 |
| Conditional model | Grade (8) | 1.02 | 0.05 | 0.37 | 0.71 |
| Conditional model | Group: Time | 1.07 | 0.08 | 0.93 | 0.35 |
| Zero model | Intercept | 1.37 | 0.30 | 1.45 | 0.15 |
| Zero model | Intervention | 1.12 | 0.21 | 0.59 | 0.55 |
| Zero model | Second measurement | 0.97 | 0.22 | -0.14 | 0.89 |
| Zero model | Gender (Girl) | 0.29 | 0.03 | -10.91 | <.001 |
| Zero model | Housing (Rental) | 1.06 | 0.22 | 0.29 | 0.77 |
| Zero model | Housing (Villa) | 0.91 | 0.16 | -0.54 | 0.59 |
| Zero model | Grade (8) | 1.02 | 0.13 | 0.14 | 0.89 |
| Zero model | Group: Time | 1.07 | 0.28 | 0.26 | 0.8 |
|  | ICC idcode:school (Intercept) | 0.99 |  |  |  |
|  | ICC school (Intercept) | 0.01 |  |  |  |
|  | Number of observations | 1390 |  |  |  |
|  | AIC | 3985.72 |  |  |  |
|  | Model R2 | 0.0197 |  |  |  |
|  | Interaction R2 (group:time) | 0.001 |  |  |  |

*Note*. The random effects were students nested in schools. Baseline for categorical variables: Time: T1; Group: control; Gender: Boy; Housing: Owned apartment, Grade: 7. Estimates are exponentiated.

**Table 14**

*Challenging Negative Thoughts Multilevel Between Subjects Model Linear Model*

| Effect | Estimate | *SE* | *t* | *df* | *p-*value |
| --- | --- | --- | --- | --- | --- |
| Intercept | 3.25 | 0.17 | 19.57 | 7.44 | <.001 |
| Group (Intervention) | -0.07 | 0.17 | -0.44 | 4.74 | 0.68 |
| Time (T2) | -0.02 | 0.09 | -0.22 | 683.53 | 0.83 |
| Gender (Girl) | -0.24 | 0.06 | -4.03 | 804.81 | <.001 |
| Housing (Rental) | 0.1 | 0.11 | 0.95 | 1197.66 | 0.34 |
| Housing (Villa) | 0.09 | 0.10 | 0.93 | 879.38 | 0.35 |
| Grade (8) | -0.43 | 0.10 | -4.35 | 33.62 | <.001 |
| Group: Time | 0.32 | 0.10 | 3.16 | 696.96 | <.001* |
| ICC idcode:school | 0.31 |  |  |  |  |
| ICC school | 0.01 |  |  |  |  |
| Number of observations | 1438 |  |  |  |  |
| AIC | 4116.21 |  |  |  |  |
| Model R2 | 0.0665 |  |  |  |  |
| Interaction R2 (group:time) | 0.0093 |  |  |  |  |

*Note*. The random effects were students nested in schools. Baseline for categorical variables: Time: T1; Group: control; Gender: Boy; Housing: Owned apartment, Grade: 7.
*Interaction remains significant after conducting a Benjamin-Hochberg FDR correction across the 13 interaction tests (adjusted *p* < .05., smallest adjusted *p* = .004).

**Table 15**

*Mobile Location while Studying Multilevel Between Subjects Cumulative Multilevel Model with a Probit Link*

| Effect | Estimate | *SE* | *Z* | *p-*value |
| --- | --- | --- | --- | --- |
| 1\|2 | -0.02 | 0.19 | -0.09 | 0.93 |
| 2\|3 | 1.69 | 0.2 | 8.32 | <.001 |
| Group (Intervention) | 0.14 | 0.18 | 0.82 | 0.41 |
| Time (T2) | -0.22 | 0.13 | -1.69 | 0.09 |
| Gender (Girl) | -0.08 | 0.09 | -0.85 | 0.39 |
| Housing (Rental) | 0.00 | 0.16 | 0.02 | 0.99 |
| Housing (Villa) | -0.03 | 0.14 | -0.24 | 0.81 |
| Grade (8) | -0.02 | 0.12 | -0.16 | 0.87 |
| Group: Time | 1.10 | 0.16 | 7.05 | <.001* |
| ICC idcode:school | 0.10 |  |  |  |
| ICC school | 0.02 |  |  |  |
| Number of observations | 1411 |  |  |  |
| AIC | 2696.53 |  |  |  |
| Model R2 | 0.0542 |  |  |  |
| Interaction R2 (group:time) | 0.0189 |  |  |  |

*Note*. The random effects were students nested in schools. Baseline for categorical variables: Time: T1; Group: control; Gender: Boy; Housing: Owned apartment, Grade: 7.
*Interaction remains significant after conducting a Benjamin-Hochberg FDR correction across the 13 interaction tests (adjusted *p* < .05., smallest adjusted *p* = .0003).

**Table 16**

*Psychological Distress (PHQ-sum) Multilevel Between Subjects Dose-Based Between Subjects with Poisson Distribution*

| Effect | Estimate | *SE* | *Z* | *p-*value |
| --- | --- | --- | --- | --- |
| Intercept | 2.53 | 0.0006 | 1531.87 | <.001 |
| Doseteach | 1.01 | 0.0006 | 9.12 | <.001 |
| Time (T2) | 1.05 | 0.0006 | 78.22 | <.001 |
| Gender (Girl) | 1.48 | 0.0006 | 648.76 | <.001 |
| Housing (Rental) | 0.91 | 0.0006 | -149.84 | <.001 |
| Housing (Villa) | 0.94 | 0.0006 | -110.14 | <.001 |
| Grade (8) | 1.15 | 0.0006 | 238.24 | <.001 |
| Doseteach:Time2 | 0.93 | 0.0006 | -116.84 | <.001 |
| ICC idcode:school | 0.97 |  |  |  |
| ICC school | 0.03 |  |  |  |
| Number of observations | 1434 |  |  |  |
| AIC | 6104.3 |  |  |  |
| Model R2 | 0.1152 |  |  |  |
| Interaction R2 (doseteach:time) | 0.0119 |  |  |  |

*Note*. The random effects were students nested in schools. The variable “Doseteach” corresponds to the number of modules completed according to teacher reports. Baseline for categorical variables: Time: T1; Group: control; Gender: Boy; Housing: Owned apartment, Grade: 7. Estimates are exponentiated.

**Table 17**

*Quality of Life (KIDSSCREEN 10) Dose-based Multilevel Between Subjects Model with Poisson Distribution*

| Effect | Estimate | *SE* | *Z* | *p-*value |
| --- | --- | --- | --- | --- |
| Intercept | 35.29 | 0.62 | 201.51 | <.001 |
| Doseteach | 1.001 | 0.003 | 0.44 | 0.66 |
| Time (T2) | 1.01 | 0.02 | 0.43 | 0.67 |
| Gender (Girl) | 0.98 | 0.01 | -2.02 | 0.04 |
| Housing (Rental) | 1.02 | 0.02 | 1.02 | 0.31 |
| Housing (Villa) | 1.03 | 0.01 | 1.78 | 0.07 |
| Grade (8) | 0.97 | 0.01 | -2.39 | 0.02 |
| Doseteach:Time 2 | 1.00 | 0.004 | -0.63 | 0.53 |
| ICC idcode:school | 0.00 |  |  |  |
| ICC school | 1.00 |  |  |  |
| Number of observations | 1401 |  |  |  |
| AIC | 8260.63 |  |  |  |
| Model R2 | 0.0101 |  |  |  |
| Interaction R2 (doseteach:time) | 0.0003 |  |  |  |

*Note*. The random effects were students nested in schools. The variable “Doseteach” corresponds to the number of modules completed according to teacher reports. Baseline for categorical variables: Time: T1; Group: control; Gender: Boy; Housing: Owned apartment, Grade: 7. Estimates are exponentiated.

**Table 18**

*Negative Consequences (GSMQ) Dose-based Multilevel Between Subjects with Gamma Distribution*

| Effect | Estimate | *SE* | *Z* | *p-*value |
| --- | --- | --- | --- | --- |
| Intercept | 1.28 | 0.06 | 4.87 | <.001 |
| Doseteach | 1.01 | 0.01 | 1.03 | 0.3 |
| Time (T2) | 1.00 | 0.02 | 0.13 | 0.9 |
| Gender (Girl) | 1.24 | 0.04 | 6.93 | <.001 |
| Housing (Rental) | 0.98 | 0.04 | -0.37 | 0.71 |
| Housing (Villa) | 1.01 | 0.03 | 0.42 | 0.67 |
| Grade (8) | 1.05 | 0.04 | 1.11 | 0.27 |
| Doseteach:time2 | 0.99 | 0.005 | -2.97 | <.001 |
| ICC idcode:school | 0.57 |  |  |  |
| ICC school | 0.00 |  |  |  |
| Number of observations | 1352 |  |  |  |
| AIC | 1380.56 |  |  |  |
| Model R2 | 0.0821 |  |  |  |
| Interaction R2 (doseteach:time) | 0.0031 |  |  |  |

*Note*. The random effects were students nested in schools. The variable “Doseteach” corresponds to the number of modules completed according to teacher reports. Baseline for categorical variables: Time: T1; Group: control; Gender: Boy; Housing: Owned apartment, Grade: 7.

**Table 19**

*Average Sleep Dose-based Multilevel Cumulative Between Subjects Model with a Log Link*

| Effect | Estimate | *SE* | *Z* | *p-*value |
| --- | --- | --- | --- | --- |
| 6\|7 | 0.01 | 0.4 | -10.42 | <.001 |
| 7\|8 | 0.18 | 0.37 | -4.58 | <.001 |
| 8\|9 | 3.04 | 0.37 | 2.98 | <.001 |
| 9\|10 | 45.73 | 0.41 | 9.43 | <.001 |
| Doseteach | 1.02 | 0.07 | 0.28 | 0.78 |
| Time (T2) | 0.79 | 0.19 | -1.22 | 0.22 |
| Gender (Girl) | 0.64 | 0.17 | -2.68 | 0.01 |
| Housing (Rental) | 0.80 | 0.29 | -0.79 | 0.43 |
| Housing (Villa) | 0.90 | 0.25 | -0.41 | 0.68 |
| Grade (8) | 0.39 | 0.25 | -3.69 | <.001 |
| Doseteach:time2 | 1.00 | 0.05 | 0 | 1 |
| ICC idcode:school | 0.50 |  |  |  |
| ICC school | 0.04 |  |  |  |
| Number of observations | 1454 |  |  |  |
| AIC | 3880.14 |  |  |  |
| Model R2 | 0.0123 |  |  |  |
| Interaction R2 (doseteach:time) | 9.08e-11 |  |  |  |

*Note*. The random effects were students nested in schools. The variable “Doseteach” corresponds to the number of modules completed according to teacher reports. Baseline for categorical variables: Time: T1; Group: control; Gender: Boy; Housing: Owned apartment, Grade: 7. Estimates are exponentiated.

**Table 20**

*Multitasking while Studying Dose-based Multilevel Cumulative Between Subjects Model with a Log Link*

| Effect | Estimate | *SE* | *Z* | *p-*value |
| --- | --- | --- | --- | --- |
| 1\|2 | 0.18 | 0.23 | -7.39 | <.001 |
| 2\|3 | 0.72 | 0.22 | -1.47 | 0.14 |
| 3\|4 | 3.15 | 0.23 | 5.07 | <.001 |
| 4\|5 | 15.99 | 0.25 | 11.21 | <.001 |
| Doseteach | 0.98 | 0.04 | -0.58 | 0.56 |
| Time (T2) | 0.97 | 0.18 | -0.18 | 0.85 |
| Gender (Girl) | 1.84 | 0.12 | 4.99 | <.001 |
| Housing (Rental) | 1.18 | 0.21 | 0.76 | 0.45 |
| Housing (Villa) | 1.13 | 0.18 | 0.67 | 0.5 |
| Grade (8) | 0.96 | 0.14 | -0.29 | 0.77 |
| Doseteach:Time 2 | 0.81 | 0.05 | -4.66 | <.001 |
| ICC idcode:school | 0.18 |  |  |  |
| ICC school | 0.01 |  |  |  |
| Number of observations | 1406 |  |  |  |
| AIC | 4251.12 |  |  |  |
| Model R2 | 0.0316 |  |  |  |
| Interaction R2 (doseteach:time) | 0.0052 |  |  |  |

*Note*. The random effects were students nested in schools. The variable “Doseteach” corresponds to the number of modules completed according to teacher reports. Baseline for categorical variables: Time: T1; Group: control; Gender: Boy; Housing: Owned apartment, Grade: 7. Estimates are exponentiated.

**Table 21**

*Avoidance Dose-based Multilevel Cumulative Between Subjects Model with a Probit Link*

| Effect | Estimate | *SE* | *Z* | *p-*value |
| --- | --- | --- | --- | --- |
| 1\|2 | -0.92 | 0.18 | -5.18 | <.001 |
| 2\|3 | 0.15 | 0.18 | 0.84 | 0.4 |
| 3\|4 | 1.17 | 0.18 | 6.51 | <.001 |
| 4\|5 | 2.16 | 0.19 | 11.35 | <.001 |
| Doseteach | 0.005 | 0.03 | 0.14 | 0.89 |
| Time (T2) | 0.08 | 0.11 | 0.79 | 0.43 |
| Gender (Girl) | 0.90 | 0.09 | 9.77 | <.001 |
| Housing (Rental) | -0.09 | 0.15 | -0.6 | 0.55 |
| Housing (Villa) | -0.06 | 0.13 | -0.44 | 0.66 |
| Grade (8) | 0.2 | 0.13 | 1.59 | 0.11 |
| Doseteach:Time 2 | -0.12 | 0.03 | -4.49 | <.001 |
| ICC idcode:school | 0.22 |  |  |  |
| ICC school | 0.01 |  |  |  |
| Number of observations | 1438 |  |  |  |
| AIC | 4209.98 |  |  |  |
| Model R2 | 0.0403 |  |  |  |
| Interaction R2 (doseteach:time) | 0.0049 |  |  |  |

*Note*. The random effects were students nested in schools. The variable “Doseteach” corresponds to the number of modules completed according to teacher reports. Baseline for categorical variables: Time: T1; Group: control; Gender: Boy; Housing: Owned apartment, Grade: 7. Estimates are exponentiated.

**Table 22**

*Negative Online Interactions Zero-Inflated Dose-based Multilevel Between Subjects Model with Truncated Poisson distribution*

| Component | Effect | Estimate | *SE* | *Z* | *p-*value |
| --- | --- | --- | --- | --- | --- |
| Conditional model | Intercept | 2.79 | 0.86 | 3.35 | <.001 |
| Conditional model | Doseteach | 0.98 | 0.05 | -0.45 | 0.65 |
| Conditional model | Time (T2) | 0.90 | 0.12 | -0.82 | 0.41 |
| Conditional model | Gender (Girl) | 0.65 | 0.16 | -1.8 | 0.07 |
| Conditional model | Housing (Rental) | 1.49 | 0.43 | 1.38 | 0.17 |
| Conditional model | Housing (Villa) | 0.59 | 0.15 | -2.01 | 0.04 |
| Conditional model | Grade (8) | 1.38 | 0.33 | 1.37 | 0.17 |
| Conditional model | Doseteach:Time 2 | 0.98 | 0.04 | -0.61 | 0.54 |
| Zero model | Intercept | 2.55 | 0.62 | 3.84 | <.001 |
| Zero model | Doseteach | 0.99 | 0.04 | -0.15 | 0.88 |
| Zero model | Time (T2) | 1.13 | 0.27 | 0.5 | 0.62 |
| Zero model | Gender (Girl) | 1.05 | 0.14 | 0.36 | 0.72 |
| Zero model | Housing (Rental) | 1.09 | 0.25 | 0.38 | 0.71 |
| Zero model | Housing (Villa) | 1.50 | 0.29 | 2.09 | 0.04 |
| Zero model | Grade (8) | 0.91 | 0.14 | -0.64 | 0.52 |
| Zero model | Doseteach:Time2 | 1.06 | 0.06 | 0.96 | 0.34 |
|  | ICC idcode (Intercept) | 0.75 |  |  |  |
|  | Number of observations | 1400 |  |  |  |
|  | AIC | 2787.57 |  |  |  |
|  | Model R2 | 0.103 |  |  |  |
|  | Interaction R2 (doseteach:time) | -0.001 |  |  |  |

*Note*. The model includes random intercepts and slopes for the effect of sex, which are nested within students (idcode) due to the lack of variation of students nested within schools. The variable “Doseteach” corresponds to the number of modules completed according to teacher reports. Baseline for categorical variables: Time: T1; Group: control; Gender: Boy; Housing: Owned apartment, Grade: 7. Estimates are exponentiated.

**Table 23**

*Daily Time on Mobile Dose-based Multilevel Between Subjects Model with Gaussian Distribution*

| Effect | Estimate | *SE* | *t* | *df* | *p-value* |
| --- | --- | --- | --- | --- | --- |
| Intercept | 3.38 | 0.18 | 18.29 | 15.91 | <.001 |
| Doseteach | 0.02 | 0.03 | 0.64 | 5.76 | 0.55 |
| Time (T2) | 0.37 | 0.11 | 3.37 | 663.54 | <.001 |
| Gender (Girl) | 0.63 | 0.10 | 6.49 | 847.77 | <.001 |
| Housing (Rental) | -0.12 | 0.16 | -0.76 | 954.24 | 0.45 |
| Housing (Villa) | -0.07 | 0.14 | -0.54 | 312.39 | 0.59 |
| Grade (8) | 0.38 | 0.13 | 3.00 | 20.1 | 0.01 |
| Doseteach:Time 2 | -0.07 | 0.03 | -2.72 | 683.37 | 0.01 |
| ICC idcode:school | 0.51 |  |  |  |  |
| ICC school | 0.01 |  |  |  |  |
| Number of observations | 1416 |  |  |  |  |
| AIC | 5122.47 |  |  |  |  |
| Model R2 | 0.0579 |  |  |  |  |
| Interaction R2 (doseteach:time) | 0.0028 |  |  |  |  |

*Note*. The random effects were students nested in schools. The variable “Doseteach” corresponds to the number of modules completed according to teacher reports. Baseline for categorical variables: Time: T1; Group: control; Gender: Boy; Housing: Owned apartment, Grade: 7.

**Table 24**

*Notifications Settings Dose-based Cumulative Multilevel Between Subjects Model with a Log Link*

| Effect | Estimate | *SE* | *Z* | *p-value* |
| --- | --- | --- | --- | --- |
| 1\|2 | 0.16 | 0.27 | -6.91 | <.001 |
| 2\|3 | 0.33 | 0.26 | -4.23 | <.001 |
| 3\|4 | 1.44 | 0.26 | 1.43 | 0.15 |
| 4\|5 | 6.10 | 0.27 | 6.81 | <.001 |
| Doseteach | 0.95 | 0.04 | -1.12 | 0.26 |
| Time (T2) | 1.09 | 0.19 | 0.47 | 0.64 |
| Gender (Girl) | 1.43 | 0.14 | 2.58 | 0.01 |
| Housing (Rental) | 0.57 | 0.24 | -2.34 | 0.02 |
| Housing (Villa) | 0.99 | 0.21 | -0.05 | 0.96 |
| Grade (8) | 0.86 | 0.17 | -0.91 | 0.36 |
| Doseteach:Time 2 | 1.01 | 0.05 | 0.26 | 0.79 |
| ICC idcode:school | 0.36 |  |  |  |
| ICC school | 0.005 |  |  |  |
| Number of observations | 1408 |  |  |  |
| AIC | 4357.3 |  |  |  |
| Model R2 | 0.0094 |  |  |  |
| Interaction R2 (Doseteach:Time 2) | 1.56e-5 |  |  |  |

*Note*. The random effects were students nested in schools. The variable “Doseteach” corresponds to the number of modules completed according to teacher reports. Baseline for categorical variables: Time: T1; Group: control; Gender: Boy; Housing: Owned apartment, Grade: 7. Estimates are exponentiated.

**Table 25**

*Breathing Strategies Dose-based Cumulative Multilevel Between Subjects Model with a Log Link*

| Effect | Estimate | *SE* | *Z* | *p-value* |
| --- | --- | --- | --- | --- |
| 1\|2 | 0.27 | 0.33 | -3.92 | <.001 |
| 2\|3 | 1.03 | 0.33 | 0.09 | 0.93 |
| 3\|4 | 5.91 | 0.33 | 5.35 | <.001 |
| 4\|5 | 36.97 | 0.35 | 10.23 | <.001 |
| Doseteach | 1.00 | 0.07 | 0.06 | 0.95 |
| Time (T2) | 1.01 | 0.18 | 0.08 | 0.94 |
| Gender (Girl) | 1.37 | 0.14 | 2.23 | 0.03 |
| Housing (Rental) | 0.95 | 0.25 | -0.19 | 0.85 |
| Housing (Villa) | 1.29 | 0.22 | 1.17 | 0.24 |
| Grade (8) | 0.84 | 0.21 | -0.8 | 0.42 |
| Doseteach:Time 2 | 1.09 | 0.05 | 1.89 | 0.06 |
| ICC idcode:school | 0.35 |  |  |  |
| ICC school | 0.03 |  |  |  |
| Number of observations | 1440 |  |  |  |
| AIC | 4358.99 |  |  |  |
| Model R2 | 0.0104 |  |  |  |
| Interaction R2 (doseteach:time) | 0.0008 |  |  |  |

*Note*. The random effects were students nested in schools. The variable “Doseteach” corresponds to the number of modules completed according to teacher reports. Baseline for categorical variables: Time: T1; Group: control; Gender: Boy; Housing: Owned apartment, Grade: 7. Estimates are exponentiated.

**Table 26**

*Talk about Problems (GHSQ) Dose-based Zero-Inflated Multilevel Between Subjects Model with Truncated Poisson Link*

| Component | Effect | Estimate | *SE* | *Z* | *p-value* |
| --- | --- | --- | --- | --- | --- |
| Conditional model | Intercept | 1.29 | 0.2 | 1.67 | 0.1 |
| Conditional model | Doseteach | 1.05 | 0.03 | 2.05 | 0.04 |
| Conditional model | Time (T2) | 1.08 | 0.14 | 0.61 | 0.54 |
| Conditional model | Gender (Girl) | 1.06 | 0.09 | 0.73 | 0.47 |
| Conditional model | Housing (Rental) | 0.95 | 0.13 | -0.35 | 0.72 |
| Conditional model | Housing (Villa) | 0.84 | 0.1 | -1.54 | 0.12 |
| Conditional model | Grade (8) | 1.11 | 0.1 | 1.1 | 0.27 |
| Conditional model | Doseteach:Time (T2) | 1.00 | 0.03 | -0.13 | 0.9 |
| Zero model | Intercept | 1.42 | 0.31 | 1.61 | 0.11 |
| Zero model | Doseteach | 1.01 | 0.04 | 0.18 | 0.85 |
| Zero model | Time (T2) | 0.98 | 0.21 | -0.1 | 0.92 |
| Zero model | Gender (Girl) | 0.29 | 0.03 | -10.91 | <.001 |
| Zero model | Housing (Rental) | 1.09 | 0.23 | 0.43 | 0.66 |
| Zero model | Housing (Villa) | 0.93 | 0.17 | -0.41 | 0.68 |
| Zero model | Grade (8) | 1.01 | 0.14 | 0.1 | 0.92 |
| Zero model | Doseteach:Time 2 | 1.01 | 0.05 | 0.22 | 0.83 |
|  | ICC idcode:school (Intercept) | 1.00 |  |  |  |
|  | ICC school (Intercept) | 6.56e-9 |  |  |  |
|  | Number of observations | 1390 |  |  |  |
|  | AIC | 4021.65 |  |  |  |
|  | Model R2 | 0.0237 |  |  |  |
|  | Interaction R2 (doseteach:time) | 1.44e-5 |  |  |  |

*Note*. The random effects were students nested in schools. The variable “Doseteach” corresponds to the number of modules completed according to teacher reports. Baseline for categorical variables: Time: T1; Group: control; Gender: Boy; Housing: Owned apartment, Grade: 7. Estimates are exponentiated.

**Table 27**

*Challenging Negative Thoughts Dose-based Multilevel Between Subjects Linear Model*

| Effect | Estimate | SE | *t* | *df* | *p-*value |
| --- | --- | --- | --- | --- | --- |
| Intercept | 3.07 | 0.13 | 22.99 | 15.8 | <.001 |
| Doseteach | -0.01 | 0.03 | -0.45 | 7.19 | 0.66 |
| Time (T2) | -0.04 | 0.08 | -0.51 | 683.65 | 0.61 |
| Gender (Girl) | -0.24 | 0.06 | -3.94 | 810.75 | <.001 |
| Housing (Rental) | 0.11 | 0.11 | 1.01 | 1132.73 | 0.32 |
| Housing (Villa) | 0.08 | 0.09 | 0.85 | 666.42 | 0.40 |
| Doseteach:Time (T2) | 0.08 | 0.02 | 3.72 | 703.74 | <.001 |
| ICC idcode:school | 0.31 |  |  |  |  |
| ICC school | 0.01 |  |  |  |  |
| Number of observations | 1438 |  |  |  |  |
| AIC | 4131.60 |  |  |  |  |
| Model R2 | 0.0342 |  |  |  |  |
| Interaction R2 (doseteach:time) | 0.0123 |  |  |  |  |

*Note*. The random effects were students nested in schools. The variable “Doseteach” corresponds to the number of modules completed according to teacher reports. Baseline for categorical variables: Time: T1; Group: control; Gender: Boy; Housing: Owned apartment.

**Table 28**

*Mobile Location while Studying Dose-based Multilevel Between Subjects Cumulative Multilevel Model with a Probit Link*

| Effect | Estimate | *SE* | *Z* | *p-*value |
| --- | --- | --- | --- | --- |
| 1\|2 | -0.03 | 0.20 | -0.13 | 0.9 |
| 2\|3 | 1.66 | 0.21 | 8.03 | <.001 |
| Doseteach | 0.03 | 0.04 | 0.68 | 0.5 |
| Time (T2) | -0.08 | 0.12 | -0.62 | 0.53 |
| Gender (Girl) | -0.07 | 0.09 | -0.84 | 0.4 |
| Housing (Rental) | 0.005 | 0.16 | 0.03 | 0.97 |
| Housing (Villa) | -0.02 | 0.14 | -0.17 | 0.87 |
| Doseteach:Time 2 | 0.20 | 0.03 | 6.25 | <.001 |
| ICC idcode:school | 0.10 |  |  |  |
| ICC school | 0.02 |  |  |  |
| Number of observations | 1411 |  |  |  |
| AIC | 2709.28 |  |  |  |
| Model R2 | 0.049 |  |  |  |
| Interaction R2 (group:time) | 0.0147 |  |  |  |

*Note*. The random effects were students nested in schools. The variable “Doseteach” corresponds to the number of modules completed according to teacher reports. Baseline for categorical variables: Time: T1; Gender: Boy; Housing: Owned apartment, Grade: 7.

**Table  29**

*Psychological Distress (PHQ-sum) Multilevel Within Subjects Model with Poisson Distribution*

| Effect | Estimate | *SE* | *Z* | *p-*value |
| --- | --- | --- | --- | --- |
| Intercept | 2.34 | 0.40 | 4.95 | <.001 |
| Time (T2) | 0.75 | 0.06 | -3.83 | <.001 |
| Time (T3) | 0.69 | 0.05 | -4.7 | <.001 |
| Gender (Girl) | 1.42 | 0.14 | 3.47 | <.001 |
| Housing (Rental) | 1.08 | 0.19 | 0.44 | 0.66 |
| Housing (Villa) | 1.08 | 0.17 | 0.47 | 0.64 |
| Idcode (Intercept) | 0.16 |  |  |  |
| Number of observations | 360 |  |  |  |
| AIC | 1425.71 |  |  |  |
| Model R² | 0.092 |  |  |  |
| Time R² | 0.045 |  |  |  |

*Note*. The random effects were random intercept for students. Baseline for categorical variables: Time: T1; Gender: Boy; Housing: Owned apartment. Estimates are exponentiated.

**Table 30**

*Quality of Life (KIDSSCREEN 10) Multilevel Within Subjects Model with Poisson Distribution*

| Effect | Estimate | *SE* | *Z* | *p-*value |
| --- | --- | --- | --- | --- |
| Intercept | 35.06 | 1.28 | 97.76 | <.001 |
| Time (T2) | 1.01 | 0.02 | 0.45 | 0.65 |
| Time (T3) | 0.98 | 0.02 | -0.75 | 0.45 |
| Gender (Girl) | 1.00 | 0.02 | 0.19 | 0.85 |
| Housing (Rental) | 1.03 | 0.04 | 0.84 | 0.4 |
| Housing (Villa) | 1.03 | 0.03 | 1 | 0.32 |
| ICC idcode (Intercept) | 7.68e-22 |  |  |  |
| Number of observations | 349 |  |  |  |
| AIC | 2036.15 |  |  |  |
| Model R2 | 0.0072 |  |  |  |
| Time R2 | 0.0043 |  |  |  |

*Note*. The random effects were random intercept for students. Baseline for categorical variables: Time: T1; Gender: Boy; Housing: Owned apartment. Estimates are exponentiated.

**Table 31**

*Negative Consequences (GSMQ) Multilevel  Within Subjects Model with Gamma Distribution*

| Effect | Estimate | *SE* | *t* | *p-*value |
| --- | --- | --- | --- | --- |
| Intercept | 0.24 | 0.09 | 2.73 | 0.01 |
| Time (T2) | -0.05 | 0.03 | -1.88 | 0.06 |
| Time (T3) | -0.05 | 0.03 | -1.74 | 0.08 |
| Gender (Girl) | 0.19 | 0.06 | 3 | <.001 |
| Housing (Rental) | 0.07 | 0.09 | 0.82 | 0.41 |
| Housing (Villa) | 0.06 | 0.07 | 0.87 | 0.39 |
| ICC idcode (Intercept) | 0.24 |  |  |  |
| Number of observations | 345 |  |  |  |
| AIC | 307.86 |  |  |  |
| Model R2 | 0.079 |  |  |  |
| Time R2 | 0.0053 |  |  |  |

*Note*. The random effects were random intercept for students. Baseline for categorical variables: Time: T1; Gender: Boy; Housing: Owned apartment.

**Table 32**

*Average Sleep Multilevel Cumulative Within Subjects Model with a Log Link*

| Effect | Estimate | *SE* | *Z* | *p-*value |
| --- | --- | --- | --- | --- |
| 6\|7 | 0.012 | 0.63 | -6.96 | <.001 |
| 7\|8 | 0.21 | 0.55 | -2.79 | 0.005 |
| 8\|9 | 2.54 | 0.55 | 1.67 | 0.094 |
| 9\|10 | 24.25 | 0.61 | 5.22 | <.001 |
| Time (T2) | 0.89 | 0.24 | -0.46 | 0.64 |
| Time (T3) | 0.67 | 0.25 | -1.55 | 0.12 |
| Gender (Girl) | 0.58 | 0.31 | -1.65 | 0.098 |
| Housing (Rental) | 1.04 | 0.56 | 0.073 | 0.94 |
| Housing (Villa) | 1.30 | 0.50 | 0.53 | 0.59 |
| ICC idcode (Intercept) | 0.35 |  |  |  |
| Number of observations | 363 |  |  |  |
| AIC | 950.98 |  |  |  |
| Model R² | 0.0099 |  |  |  |
| Time R² | 0.0028 |  |  |  |

*Note*. The random effects were random intercept for students. Baseline for categorical variables: Time: T1; Gender: Boy; Housing: Owned apartment. Estimates are exponentiated.

**Table 33**

*Multitasking while Studying Multilevel Cumulative Within Subjects Model with a Log Link*

| Effect | Estimate | *SE* | *Z* | *p-*value |
| --- | --- | --- | --- | --- |
| 1\|2 | 0.19 | 0.55 | -2.99 | 0.003 |
| 2\|3 | 0.83 | 0.53 | -0.33 | 0.74 |
| 3\|4 | 4.22 | 0.54 | 2.65 | 0.008 |
| 4\|5 | 19.47 | 0.57 | 5.18 | <.001 |
| Time (T2) | 0.65 | 0.24 | -1.72 | 0.085 |
| Time (T3) | 0.40 | 0.25 | -3.65 | <.001 |
| Gender (Girl) | 2.47 | 0.33 | 2.74 | 0.006 |
| Housing (Rental) | 1.08 | 0.55 | 0.14 | 0.88 |
| Housing (Villa) | 1.05 | 0.47 | 0.10 | 0.91 |
| ICC idcode (Intercept) | 0.38 |  |  |  |
| Number of observations | 357 |  |  |  |
| AIC | 1078.36 |  |  |  |
| Model R² | 0.025 |  |  |  |
| Time R² | 0.012 |  |  |  |

*Note*. The random effects were random intercept for students. Baseline for categorical variables: Time: T1; Gender: Boy; Housing: Owned apartment. Estimates are exponentiated.

**Table 34**

*Avoidance Multilevel Cumulative Within Subjects Model with a Probit Link*

| Effect | Estimate | *SE* | *Z* | *p-*value |
| --- | --- | --- | --- | --- |
| 1\|2 | -1.04 | 0.35 | -3.00 | 0.003 |
| 2\|3 | 0.008 | 0.34 | 0.02 | 0.98 |
| 3\|4 | 0.9 | 0.34 | 2.84 | 0.005 |
| 4\|5 | 2.09 | 0.36 | 5.78 | <.001 |
| Time (T2) | -0.52 | 0.14 | -3.76 | <.001 |
| Time (T3) | -0.71 | 0.15 | -4.81 | <.001 |
| Gender (Girl) | 0.75 | 0.21 | 3.63 | <.001 |
| Housing (Rental) | 0.15 | 0.35 | 0.43 | 0.67 |
| Housing (Villa) | 0.03 | 0.30 | 0.10 | 0.92 |
| ICC idcode (Intercept) | 0.20 |  |  |  |
| Number of observations | 360 |  |  |  |
| AIC | 1039.01 |  |  |  |
| Model R² | 0.041 |  |  |  |
| Time R² | 0.024 |  |  |  |

*Note*. The random effects were random intercept for students. Baseline for categorical variables: Time: T1; Gender: Boy; Housing: Owned apartment.

**Table 35**

*Negative Online Interactions Zero-Inflated Multilevel Within Subjects Model with Truncated Poisson distribution*

| Component | Effect | Estimate | *SE* | *Z* | *p-value* |
| --- | --- | --- | --- | --- | --- |
| Conditional | Intercept | 6.26 | 5.08 | 2.26 | 0.02 |
| Conditional | Time (T2) | 0.66 | 0.17 | -1.56 | 0.12 |
| Conditional | Time (T3) | 1.16 | 0.26 | 0.67 | 0.5 |
| Conditional | Gender (Girl) | 0.30 | 0.12 | -2.94 | <.001 |
| Conditional | Housing (Rental) | 0.56 | 0.48 | -0.68 | 0.5 |
| Conditional | Housing (Villa) | 0.42 | 0.34 | -1.07 | 0.28 |
| Zero | Baseline | 6.38 | 4.03 | 2.94 | <.001 |
| Zero | Time (T2) | 1.88 | 0.60 | 1.96 | 0.05 |
| Zero | Time (T3) | 1.86 | 0.60 | 1.92 | 0.05 |
| Zero | Housing (Rental) | 0.30 | 0.20 | -1.83 | 0.07 |
| Zero | Housing (Villa) | 0.43 | 0.27 | -1.34 | 0.18 |
|  | ICC idcode (Intercept) | 0.89 |  |  |  |
|  | Number of observations | 358 |  |  |  |
|  | AIC | 675.72 |  |  |  |
|  | Model R2 | 0.23 |  |  |  |
|  | Time R2 | 0.0015 |  |  |  |

*Note*. The random effects were random intercept for students. Baseline for categorical variables: Time: T1; Gender: Boy; Housing: Owned apartment. Estimates are exponentiated.

**Table 36**

*Daily Time on Mobile Multilevel Within Subjects Linear Model*

| Effect | Estimate | *SE* | *t* | *df* | *p-*value |
| --- | --- | --- | --- | --- | --- |
| Intercept | 3.00 | 0.37 | 8.2 | 282.68 | <.001 |
| Time (T2) | 0.21 | 0.16 | 1.34 | 236.39 | 0.18 |
| Time (T3) | 0.41 | 0.16 | 2.62 | 235.84 | 0.01 |
| Gender (Girl) | 0.54 | 0.23 | 2.39 | 130.78 | 0.02 |
| Housing (Rental) | 0.43 | 0.38 | 1.14 | 284.81 | 0.26 |
| Housing (Villa) | 0.18 | 0.33 | 0.55 | 310.61 | 0.59 |
| idcode (Intercept) | 0.52 |  |  |  |  |
| Number of observations | 360 |  |  |  |  |
| AIC | 1321.48 |  |  |  |  |
| Model R² | 0.039 |  |  |  |  |
| Time R² | 0.011 |  |  |  |  |

*Note*. The random effects were random intercept for students.

**Table 37**

*Notifications Settings Cumulative Multilevel Within Subjects Model with a Probit Link*

| Effect | Estimate | *SE* | *Z* | *p-*value |
| --- | --- | --- | --- | --- |
| 1\|2 | -0.90 | 0.39 | -2.34 | 0.02 |
| 2\|3 | -0.43 | 0.38 | -1.12 | 0.26 |
| 3\|4 | 0.67 | 0.38 | 1.76 | 0.08 |
| 4\|5 | 1.51 | 0.39 | 3.93 | <.001 |
| Time (T2) | 0.39 | 0.15 | 2.61 | 0.009 |
| Time (T3) | 0.41 | 0.15 | 2.76 | 0.006 |
| Gender (Girl) | 0.51 | 0.25 | 2.04 | 0.04 |
| Housing (Rental) | -0.36 | 0.39 | -0.91 | 0.36 |
| Housing (Villa) | -0.04 | 0.33 | -0.11 | 0.91 |
| ICC idcode (Intercept) | 0.57 |  |  |  |
| Number of observations | 356 |  |  |  |
| AIC | 1026.88 |  |  |  |
| Model R² | 0.023 |  |  |  |
| Time R² | 0.0095 |  |  |  |

*Note*. The random effects were random intercept for students. Baseline for categorical variables: Time: T1; Gender: Boy; Housing: Owned apartment.

**Table 38**

*Breathing Strategies Cumulative Multilevel Within Subjects Model with a Log Link*

| Effect | Estimate | *SE* | *Z* | *p-*value |
| --- | --- | --- | --- | --- |
| 1\|2 | 0.45 | 0.63 | -1.25 | 0.21 |
| 2\|3 | 1.92 | 0.63 | 1.04 | 0.30 |
| 3\|4 | 15.77 | 0.65 | 4.24 | <.001 |
| 4\|5 | 156.27 | 0.71 | 7.15 | <.001 |
| Time (T2) | 1.99 | 0.25 | 2.71 | 0.007 |
| Time (T3) | 1.45 | 0.25 | 1.49 | 0.14 |
| Gender (Girl) | 2.80 | 0.40 | 2.57 | 0.01 |
| Housing (Rental) | 1.63 | 0.65 | 0.76 | 0.45 |
| Housing (Villa) | 2.52 | 0.55 | 1.67 | 0.095 |
| ICC idcode (Intercept) | 0.52 |  |  |  |
| Number of observations | 360 |  |  |  |
| AIC | 1038.34 |  |  |  |
| Model R² | 0.022 |  |  |  |
| Time R² | 0.0072 |  |  |  |

*Note*. The random effects were random intercept for students. Baseline for categorical variables: Time: T1; Gender: Boy; Housing: Owned apartment. Estimates are exponentiated.

**Table 39**

*Talk about Problems (GHSQ) Zero-Inflated Multilevel Within Subjects Model with Truncated Poisson Link*

| Component | Effect | Estimate | *SE* | *Z* | *p-*value |
| --- | --- | --- | --- | --- | --- |
| Conditional | Intercept | 2.42 | 0.57 | 3.79 | <.001 |
| Conditional | Time (T2) | 1.15 | 0.18 | 0.88 | 0.38 |
| Conditional | Time (T3) | 1.02 | 0.17 | 0.14 | 0.89 |
| Conditional | Gender (Girl) | 0.91 | 0.13 | -0.69 | 0.49 |
| Conditional | Housing (Rental) | 0.96 | 0.20 | -0.22 | 0.83 |
| Conditional | Housing (Villa) | 0.53 | 0.11 | -3.18 | <.001 |
| Zero | Intercept | 1.51 | 0.65 | 0.95 | 0.34 |
| Zero | Time (T2) | 0.86 | 0.23 | -0.57 | 0.57 |
| Zero | Time (T3) | 1.05 | 0.28 | 0.19 | 0.85 |
| Zero | Gender (Girl) | 0.42 | 0.09 | -3.91 | <.001 |
| Zero | Housing (Rental) | 0.91 | 0.39 | -0.22 | 0.83 |
| Zero | Housing (Villa) | 0.94 | 0.36 | -0.16 | 0.87 |
|  | ICC idcode (Intercept) | 0.75 |  |  |  |
|  | Number of observations | 353 |  |  |  |
|  | AIC | 1009.17 |  |  |  |
|  | Model R2 | 0.1725 |  |  |  |
|  | Time R2 | -0.0002 |  |  |  |

*Note*. The random effects were random intercept for students. Baseline for categorical variables: Time: T1; Gender: Boy; Housing: Owned apartment. Estimates are exponentiated.

**Table 40**

*Challenging Negative Thoughts Multilevel Within Subjects Linear Model*

| Effect | Estimate | *SE* | *t* | *df* | *p-*value |
| --- | --- | --- | --- | --- | --- |
| Intercept | 2.86 | 0.24 | 11.99 | 243.98 | <.001 |
| Time (T2) | 0.35 | 0.12 | 2.94 | 238.89 | <.001 |
| Time (T3) | 0.25 | 0.12 | 2.12 | 238.18 | 0.04 |
| Gender (Girl) | -0.14 | 0.13 | -1.03 | 127.12 | 0.3 |
| Housing (Rental) | 0.46 | 0.24 | 1.9 | 223.19 | 0.06 |
| Housing (Villa) | 0.30 | 0.22 | 1.38 | 248.2 | 0.17 |
| idcode (Intercept) | 0.20 |  |  |  |  |
| Number of observations | 360 |  |  |  |  |
| AIC | 1054.03 |  |  |  |  |
| Model R² | 0.037 |  |  |  |  |
| Time R² | 0.020 |  |  |  |  |

*Note*. The random effects were random intercept for students. Baseline for categorical variables: Time: T1; Gender: Boy; Housing: Owned apartment.

**Table 41**

*Mobile Location while Studying Multilevel Within Subjects Cumulative Multilevel Model with a Probit Link*

| Effect | Estimate | *SE* | *Z* | *p-*value |
| --- | --- | --- | --- | --- |
| 1\|2 | -0.55 | 0.38 | -1.46 | 0.15 |
| 2\|3 | 1.67 | 0.40 | 4.20 | <.001 |
| Time (T2) | 0.55 | 0.17 | 3.29 | 0.001 |
| Time (T3) | 0.61 | 0.17 | 3.64 | <.001 |
| Gender (Girl) | -0.06 | 0.24 | -0.25 | 0.80 |
| Housing (Rental) | 0.09 | 0.39 | 0.23 | 0.81 |
| Housing (Villa) | -0.17 | 0.34 | -0.49 | 0.63 |
| ICC idcode (Intercept) | 0.24 |  |  |  |
| Number of observations | 359 |  |  |  |
| AIC | 660.78 |  |  |  |
| Model R² | 0.034 |  |  |  |
| Time R² | 0.025 |  |  |  |

*Note*. The random effects were random intercept for students. Baseline for categorical variables: Time: T1; Gender: Boy; Housing: Owned apartment.
